# Exosomes From Adipose-Derived Stem Cells: The Emerging Roles and Applications in Tissue Regeneration of Plastic and Cosmetic Surgery

**DOI:** 10.3389/fcell.2020.574223

**Published:** 2020-09-10

**Authors:** Mingchen Xiong, Qi Zhang, Weijie Hu, Chongru Zhao, Wenchang Lv, Yi Yi, Yiping Wu, Min Wu

**Affiliations:** Department of Plastic Surgery, Tongji Hospital, Tongji Medical College, Huazhong University of Science and Technology, Wuhan, China

**Keywords:** adipose-derived stem cells, exosomes, tissue regeneration, biological activity, function

## Abstract

Adipose-derived stem cells (ASCs) are an important stem cell type separated from adipose tissue, with the properties of multilineage differentiation, easy availability, high proliferation potential, and self-renewal. Exosomes are novel frontiers of intercellular communication regulating the biological behaviors of cells, such as angiogenesis, immune modulation, proliferation, and migration. ASC-derived exosomes (ASC-exos) are important components released by ASCs paracrine, possessing multiple biological activities. Tissue regeneration requires coordinated “vital networks” of multiple growth factors, proteases, progenitors, and immune cells producing inflammatory cytokines. Recently, as cell-to-cell messengers, ASC-exos have received much attention for the fact that they are important paracrine mediators contributing to their suitability for tissue regeneration. ASC-exos, with distinct properties by encapsulating various types of bioactive cargoes, are endowed with great application potential in tissue regeneration, mechanically via the migration and proliferation of repair cells, facilitation of the neovascularization, and other specific functions in different tissues. Here, this article elucidated the research progress of ASC-exos about tissue regeneration in plastic and cosmetic surgery, including skin anti-aging therapy, dermatitis improvement, wound healing, scar removal, flap transplantation, bone tissue repair and regeneration, obesity prevention, fat grafting, breast cancer, and breast reconstruction. Deciphering the biological properties of ASC-exos will provide further insights for exploring novel therapeutic strategies of tissue regeneration in plastic and cosmetic surgery.

## Introduction

Adipose tissue provides a major energy storage depot for the body. Over the past years, adipose tissue has been considered as a multifunctional organ that controls metabolic homeostasis, immunity, and satiety (Yang et al., 2018). Adipose tissue can be broadly separated into fat-storing adipocytes and the adipose tissue stromal vascular fraction (SVF). SVF is a heterogeneous cell group that is traditionally isolated using enzymes such as collagenase. SVF is an easily accessible system comprised of various immune cells, erythrocytes, endothelial cells, and adipose-derived stem cells (ASCs), with the exception of adipocytes (Kim and Lee, 2020). ASCs are defined as a subset of mesenchymal stem cells (MSCs) isolated from the SVF within adipose tissue by enzymatic digestion (Gentile et al., 2019b). ASCs are specifically valuable because they can be easily harvested with the properties of abundance and convenient separation. In terms of cell identification, ASCs exhibit a mesenchymal-like morphology and the expression profile of CD34^+^, CD44^+^, CD31^–^, and CD45^–^ cell surface markers (Shukla et al., 2020). ASCs are not only precursors to adipocytes but also multipotent progenitors to a variety of cells including osteoblasts, chondrocytes, myocytes, epithelial cells, and neuronal cells. Furthermore, ASCs possess several unique characteristics, including easy availability, high proliferation potential, self-renewal, and secretion of trophic factors and extracellular vesicles (EVs), thus offering a feasible and valid alternative to other sources of MSCs, such as bone marrow-MSCs (BMSCs) (Mazini et al., 2019). The secreted factors derived from ASCs, such as growth factors and cytokines, are known to exert paracrine signals responsible for chemoattractant, angiogenic, and prosurvival effects required for tissue regeneration (Bajek et al., 2016). ASCs are particularly useful as they can be easily harvested with minimal donor site morbidity and have a differentiation potential similar to other MSCs. Thus, ASCs have been successfully proposed as a prominent candidate in the development of tissue engineering products. As well, plastic and cosmetic surgery is a growing field uniquely positioned for the application of ASCs (Zuk et al., 2001).

The term “exosome” specifically defines a small subset of extracellular vesicles that range from 50 nm to 200 nm, which have been found in numerous body fluids, including blood, urine, cerebrospinal fluid, breast milk, saliva, lymph, and bile, under both healthy and pathological conditions (Wang et al., 2018). Exosomes are formed when the endosome membrane invaginates to produce a multivesicular body which upon fusing with the cell membrane to release the vesicles within as exosomes into the extracellular space (Toh et al., 2018). Consequently, the key expression biomarkers of exosomes, which are generally recognized as exosome-associated characteristics, are proteins associated with endocytosis and endosomal traffickings such as tetraspanins (CD81, CD63, CD9), ALIX, TSG101, caveolins, clathrin, and transferrin receptors, due to the different mechanisms of secretion (Hong P. et al., 2019). Exosomes are packed with cell-type-specific combinations of proteins (cytoskeletal proteins, transmembrane proteins, and heat shock proteins), nucleic acids (DNA, mRNA, miRNA, long and short non-coding RNA), lipids, and enzymes (GAPDH, ATPase, pgk1), shuttling these active cargoes between different cells involving in a complex intercellular communication system. These cargoes are wrapped in the membrane to protect from degradation and transport to the surrounding cells (Kalluri and LeBleu, 2020). Thus, exosomes have some special biological characteristics and processes and are capable of potentially modulating the specific activity of the recipient target cells.

The important aim of plastic and cosmetic field is closely associated with tissue regeneration, and to repair the morphology and function of congenital or acquired defects through many treatment methods, including medical imaging, microsurgery, composite tissue allotransplantation, nanotechnology, cell biology, and biomaterials (Naderi et al., 2017). Adipose tissue possesses important functions in immune modulation, wound healing, and tissue regeneration. Nowadays, autologous adipose tissue application is explored to be applied in improving skin quality, contour irregularities, wound repair and soft tissue regeneration in plastic and cosmetic surgery (Strong et al., 2019). Its efficacy and safety are widely accepted, but there is a lack of universally recognized mechanisms. However, given that the characteristics of adipose tissue vary dramatically depending on the donor status, the effect is of individual difference (Wang et al., 2013). Besides, significantly altering the components or biomechanical properties of the adipose tissue, such as by removing stromal cells from the adipose tissue, will subject to more complex applications. Therefore, autologous adipose tissue cannot be easily used as a drug. Thus, ASCs and ASC-exos are very important derivatives from fat tissue, capturing intensive attention. Exosomes carry specific contents of the parental ASCs, including DNAs, RNAs, lipids, cytokines, enzymes. Exosomes are capable of protecting their cargoes from degradation and are highly stable in serum and blood, thus efficiently delivering cargoes to target cells. ASC-exos and ASCs perform their functions via different mechanisms. The ASC-exos are used as tools for repairing and regenerated activation of damaged cells, and are now considered to orchestrate the events required for tissue regeneration, immune function, tissue homeostasis and development of cell fate. Hence, although without differentiation ability, ASC-exos can mimic the capacity of ASCs for innovative cell-free therapy, such as tissue regeneration and repair, reduction of injuries, and anti-inflammation. In terms of storage and delivery, unlike ASCs, exosomes are small non-living substances that can be sterile filtered and frozen without cryo-preservatives. From manufacturing and storage to delivery, there is no need to maintain cell viability and function. ASC-exos have certain advantages over ASCs in production, storage, shelf life, delivery, and potentially ready-to-use biological products (Vizoso et al., 2017). Moreover, ASC-exos are potentially safer therapeutic agents than ASCs. Compared with ASCs, ASC-exos might avoid cell therapy-associated problems, including limited cell survival, immune rejection efficacy, senescence-induced genetic instability, inactivate function, and the possibility of unfavorable differentiation (Figueroa et al., 2014). Owing to their multiple features, ASC-exos have shown therapeutic potential in many clinical diseases, especially in tissue regeneration, such as skin repairing, fat grafting, and various reconstruction operation. In addition, exosomes have been innovatively utilized for targeted drug delivery and as gene carriers for regenerative medicine (Mehryab et al., 2020). But actually, ASC-exos lack enough clinical trials to confirm the safety and effectiveness (Table 1).

**TABLE 1:** Characteristic comparison of ASC-exos and ASCs.

	ASC-exos	ASCs
Source	acquisition from ASCs with exosome separation methods, exist in adipose tissue and stable in serum and blood	easy acquisition and high yield from adipocyte tissues, especially from white adipose tissue
Morphology	small lipid bilayer vesicles	mesenchymal-like cells
Management	could be sterile filtered and frozen without cryo-preservatives, easily long-term storage and delivery, easily keep biological activity	should preserve cell viability and function from manufacture to storage and delivery, high storage requirements, complex cultivation
Biological properties	protect cargoes from degradation, target specificity, good tissue permeability, intercellular communication, immune function, tissue homeostasis and development of cell fate	multi-lineage differentiation, secret great kinds of growth factors by paracrine function, prosurvival effects, regulation of immune function, angiogenesis
Secretome	DNAs, RNAs, lipids, cytokines, enzymes from the parent cell	exosomes, cytokines, DNAs, RNAs, lipids, enzymes
Biosafety	limited immunogenicity, high biosafety	immunogenicity, biosafety
Applications	considered as multiple bioactive substances for tissue regeneration, could be gene modification, upload drugs as carriers, upload in other carriers such as nanomaterials	considered as ideal stem cell source for cell and tissue regeneration; could be gene modification, upload in carriers such as nanomaterials
Clinical trials	lack enough clinical trials to confirm the safety and effectiveness	security and effectiveness are verified in many diseases
Application disadvantages	relative low purity and yield, complicated components, substantial degree of heterogeneity in dosing regimens in the reported cases, lacking *in vivo* clinical trials	limited cell survival, immune rejection efficacy, senescence-induced genetic instability, inactivate function, and the possibility of unfavorable differentiation, individual differences

*Abbreviations: ASCs, Adipose-derived stem cells; ASC-exos, ASC-derived exosomes.*

In this review, we mainly summarize the latest research about the functions and investigations of ASC-exos concerned with tissue regeneration in plastic and cosmetic surgery, including skin anti-aging therapy, dermatitis improvement, wound healing, scar removal, flap transplantation, bone tissue repair and regeneration, obesity prevention, fat grafting, breast cancer, and breast reconstruction. We hope this will provide further insights into the pivotal roles and applications of ASC-exos in tissue engineering and regenerative therapies. For these goals, we searched the adipose-derived stem cells/ASCs, exosomes and the above 8 related fields in plastic and cosmetic surgery as keywords on Pubmed in the recent 5 years. These searched studies involved in cell, animal experiments, or clinical trials, especially the original articles were included according to relevance to the topic.

## ASC-exos in Skin Aging

The skin is subject to an unpreventable intrinsic aging process, along with the exogenous factors-induced aging state. Particularly, ultraviolet radiation results in premature skin aging, also known as extrinsic skin aging or photoaging. The most typical features of skin aging are the loss of elasticity and the generation of wrinkles, which are attributed to the structural and functional changes in skin cells and tissues (Li et al., 2019). Among skin cells, human dermal fibroblasts (HDFs) and keratinocytes (HaCaTs) are regarded as barriers to prevent skin from time aging and ultraviolet B (UVB) photoaging. Various improvement strategies, such as antioxidants, retinoids, peptides, growth factors, and autologous patient fat or collagen graft, have been used to fight against skin aging (Kim et al., 2019). Because of the poor penetration through the stratum and short-term maintenance for several months, these above strategies are now not ideal. It is hopeful to reduce skin aging pinned on ASCs and ASC-derivatives, which could regulate HDFs proliferation, migration, and collagen expression.

**FIGURE 1:**
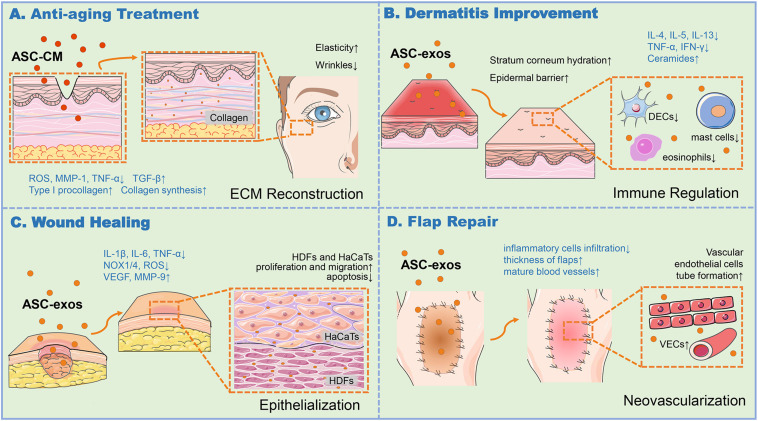
ASC-exos function in various skin associated applications. **(A)** ASC-CM and BMSC-exos could produce ROS at a low level, downregulate TNF-α, upregulate TGF-β to increase MMP-1 and procollagen type I expression for collagen synthesis, thus enhancing the skin elasticity and ease the wrinkles for anti-aging. **(B)** ASC-exos was capable to enhance stratum corneum hydration, reduce the secretion of inflammatory cytokines such as IL-4, IL-5, IL-13, IFN-γ, and TNF-α, and alleviate the infiltration of mast cells, dendritic epidermal cells (DECs) in skin lesions and eosinophils in the blood, and produce ceramides to restore the epidermal barrier, thus relieving the dermatitis of skin. **(C)** ASC-exos reduced the production of ROS, decrease the expression of IL-6, IL-1β, TNF-α, and the oxidative stress-related proteins such as NADPH oxidase 1/4 (NOX1/4), increase MMP-9 and VEGF to ameliorate ECM reconstruction, thus fostering HDFs proliferation and migration to reinforce the re-epithelialization. **(D)** ASC-exos was conducive to promote tube formation of VECs, increase tissue thickness, and reduce the infiltration of inflammatory cells to relieve the inflammation and apoptosis for the high survival rate of the skin flap. ASCs, Adipose-derived stem cells; ASC-exos, ASC-derived exosomes; HDFs, Human Dermal Fibroblasts; HaCaTs, Human Keratinocytes; ECM, Extracellular Matrix; ROS, Reactive Oxygen Species; MMP-1/9, Matrix Metalloproteinase 1/9; IFN-γ, Interferon Gamma; TNF-α, Tumor Necrosis Factor Alpha; TGF-β, Transforming Growth Factor Beta; IL-4/5/6/13, Interleukin 4/5/6/13; NOX-1/4, NADPH Oxidase 1/4; VEGF, Vascular Endothelial Growth Factor; VECs, Vascular Endothelial Cells, VECs.

ASC-conditioned medium (ASC-CM) and ASC-exos, both containing key cytokines and growth factors secreted by the ASCs, could facilitate the regeneration and repair of various tissues and organs to exert influences on anti-oxidation, anti-wrinkle, and whitening skin. ASC-CM has been proved to protect HDFs from oxidative stress *in vitro* (Kim et al., 2008). Li et al. (2019) found that in UVB irradiation *in vitro* model, ASC-CM could effectively down-regulate the activation and transcription of UVB-induced signaling pathways such as mitogen-activated protein kinases (MAPKs), activator protein 1 (AP-1), and nuclear factor kappa B (NF-κB), and up-regulate the expression of antioxidant response elements such as phase II gene HO-1 and transforming growth factor-beta (TGF-β), while reducing interleukin 6 (IL-6) secretion. Thereby ASC-CM showed a positive effect on protecting HDFs and HaCaTs from UVB-induced photoaging damage. The platelet-derived growth factor AA (PDGF-AA) contained in ASC-CM also could activate the phosphatidylinositol-4,5-bisphosphate 3-kinase (PI3K)/protein kinase B (AKT) signal pathway, and mediate photoaging-induced HDFs proliferation, extracellular matrix (ECM) deposition and remodeling in the *in vitro* experiment, which was reported by Guo et al. (2020) group. It demonstrated that the well-prepared ASC-CM played a positive role in preventing HDFs from intrinsic and extrinsic aging damages to a certain degree. Meanwhile, the result also clarified that the PDGF-AA might contribute to better outcomes with some other components of ASC-CM. However, the ingredients in ASC-CM are rather complex to synergistically achieve the anti-aging goal. The exosomes are important components in ASC-CM, might possess a positively independent or synergistic roles. Hu et al. (2019) showed that exosomes from three-dimensional cultured HDF spheroids (3D-HDF-exos) and BMSC-exos could both down-regulate tumor necrosis factor alpha (TNF-α) and up-regulated TGF-β expression, resulting in decreased matrix metalloproteinase 1 (MMP-1) and increased type I procollagen *in vitro* and a nude mouse photoaging model. These results indicated that the exosome-containing 3D-HDF-exos and BMSC-exos both had anti-skin-aging properties and the potential to prevent and treat cutaneous aging (Figure 1A).

At present, the existing research of ASC-exos on skin aging is limited. As the epidermis layer is 50–120 μm and the epidermis-dermis thickness is 2–5 mm in humans, the local treatment with exosomes can arrive at the epidermis and be absorbed on human skin (Xu et al., 2018). Exosomes derived from human stem cells, such as ASCs, are of multiple bioactive functions for skin aging treatment, deserving further research.

## ASC-exos in Atopic Dermatitis

Atopic dermatitis (AD) is a chronic inflammatory skin disease accompanied with pruritus, erythema, edema, excoriation, and thickening of the skin, leading to decreased unaesthetic appearance of skin (Lee et al., 2018). Both defective skin barrier and abnormal immune responses are crucial factors in AD development. Therefore AD with immunologic abnormalities could be treated by different or multi-pronged approaches focused on reducing the severity and frequency of symptoms, such as dietary management, drug therapy, and ultraviolet assisted therapy (Park et al., 2019). Several studies have demonstrated that allergic progress in AD could be suppressed by BMSCs and ASCs while modulating multiple targets (Shin et al., 2017). For instance, Sah et al. (2018) found that superoxide dismutase 3-transduced MSCs ameliorated AD pathology and enhanced the efficacy of MSC therapy by controlling activated immune cells, reducing expression levels of pro-inflammatory mediators in the skin of AD mice.

Nevertheless, given that AD chronic and recurrent characteristics, the therapeutic utilization of MSCs has several drawbacks, such as poor engraftment efficiency, undesired immune responses, short half-life, and difficulties in quality control (Lou et al., 2017). Exosomes are involved in the development and prognosis of AD skin diseases, including repairing leaky skin barriers as well as suppressing skin inflammation (Alves et al., 2016). Cho et al. (2018) established an AD model of NC/NGA mice treated with house dust mite antigens. In this mouse model, ASC-exos were found to ameliorate pathological symptoms such as the levels of serum IgE, the number of eosinophils in the blood, and the infiltration of mast cells, dendritic epidermal cells in skin lesions. The study suggested the immune regulation role of ASC-exos in AD. In the latter study, the same group also found that the subcutaneous injection of ASC-exos in an oxazolone-induced dermatitis model remarkably reduced trans-epidermal water loss, and enhanced stratum corneum hydration and markedly decreased the levels of inflammatory cytokines such as IL-4, IL-5, IL-13, TNF-α, interferon gamma (IFN-γ), IL-17 and TSLP, all in a dose-dependent manner (Shin et al., 2020). Interestingly, ASC-exos also induced the production of ceramides and dihydroceramides to promote skin barrier restoration (Shin et al., 2020). These studies suggested that the systemic administration of ASC-exos ameliorated AD-like symptoms through the regulation of inflammatory responses and the potential of effectively restoring epidermal barrier functions in AD (Figure 1B). ASC-exos could be a promising cell-free candidate to currently limited treatment options for AD.

## ASC-exos in Wound and Scar

Many exposed, unsightly, or chronic wounds, such as diabetic ulcers, are difficult to heal, not only causing functional disabilities but also affecting mental health. Poor wound healing eventually leads to hypertrophic scars or keloid formation, pigmentation, prolonged healing, and ulcerative skin defects (Eming et al., 2014). There are many traditional treatment methods for skin and soft tissue trauma, such as low-intensity lasers, advanced treatment dressings, negative pressure wound treatment, hyperbaric oxygen, and skin transplantation (Bellei et al., 2018). However, as wound healing is a complicated process referring to multiple cell types, growth factors, and extracellular matrix, some traditional treatments just play an auxiliary role accompanied by undesirable healing.

ASCs and SVF can produce abundant secretome groups, leading to cell proliferation and differentiation, migration, and healing microenvironment. The migration and functions of ASCs could be enhanced via PI3K/AKT pathway activated by integrin β1, resulted in the improved chronic refractory wound (Wang J. et al., 2020). Interestingly, in the wound mouse model, Bi et al. (2019) found that both SVF and human ASCs improved the function of endotheliocytes and fibroblasts, regulated gene expression, and jointly promoted skin healing. This study showed that SVF could replace ASCs for wound healing, due to the convenience of SVF applications. In the burn wound model, only autologous ASCs, but not allogeneic ASCs, significantly improved healing in acute burn wounds of the rat (Chang et al., 2018). Fujiwara et al. (2020) also constructed an ovine burn model and proved that ASCs improved grafted burn wound healing by promoting blood flow and vascular endothelial growth factor (VEGF) expression. Besides, the localized injection of ASCs could accelerate and enhance the closure of pressure ulcers (Xiao et al., 2019). Likewise, Bukowska et al. (2020) systematically ensured the safety of human SVF when injected into a murine pressure ulcer injury model. This healing function usually depends on the trophic factors of ASCs and SVF, including cytokines, growth factors, and chemokines. Notably, exosomes containing secretome from ASCs have opened the way to a newly emerging cell-free therapy.

Studies have shown that ASC-exos played a positive role in cutaneous wound healing by means of acting on HDFs and HaCaTs and other main target cells through various signal channels (Figure 1C; Qiu et al., 2020). Ma et al. (2019) exposed HaCaTs to hydrogen peroxide (H_2_O_2_) for establishing a skin lesion model, discovering that ASC-exos could foster HaCaTs proliferation, migration, and inhibit apoptosis through Wnt/β-catenin signaling pathway. Likewise, He et al. (2020) also confirmed that MALAT1-containing ASC-exos improved wound healing by targeting miR-124 and activating Wnt/β-catenin pathway. In addition, ASC-exos might also promote and optimize collagen synthesis via upregulating PI3K/Akt pathway during cutaneous wound healing (Zhang et al., 2018). Li X. et al. (2018) found that exosomes from NF-E2-related factor 2 (Nrf2)-overexpressing ASCs significantly reduced the ulcer area in the feet of diabetic rats, by promoting the proliferation and angiogenesis of endothelial cells, improving levels of senescence marker protein 30 (SMP30) and VEGF and vascular endothelial growth factor receptor 2 (VEGFR2) phosphorylation to accelerate the wound healing, as well as inhibiting reactive oxygen species (ROS) production and inflammatory cytokine expressions, such as IL-1β, IL-6, and TNF-α. Notwithstanding this superiority, exosome-based therapy of wound healing still faces the challenges of rapid clearance rate and relatively short half-life *in vivo* (Liu et al., 2017). The sustained release and retention of exosomes in the target area is an important factor for healing. Liu et al. showed hyaluronic acid (HA) might serve as exosomes immobilizer and wound dressing for durable exosomes retention at wound sites to effectively reparative effect. ASC-exos combined with HA was able to activate the HDFs activity of the wound surface and reinforced the re-epithelialization and vascularization of the wound surface (Liu et al., 2019). Moreover, several studies have confirmed the role of ASC-exos miRNAs in skin healing. For instance, Yang et al. (2020) found that highly expressed miRNA-21 derived from ASC-exos could enhance the migration and proliferation of the HaCaTs, by increasing the matrix metalloproteinase 9 (MMP-9) expression through the PI3K/AKT pathway. The overexpressing miRNA-21 could also enhance collagen synthesis and optimize collagen deposition, significantly improve the healing effect of full-thickness skin wounds in mice (Yang et al., 2020). Shi et al. (2020) verified that mmu_circ_0000250-modified ASCs derived exosomes promoted wound healing in diabetic mice by inducing miR-128-3p/SIRT1-mediated autophagy.

Compared to the single factor therapy, MSCs application is also superior in scar removal due to the MSCs-secreted various inflammatory modulators. In the rabbit scar model, Liu et al. (2014) locally injected BMSCs to regulate inflammation and prevented hypertrophic scar formation, attributing to BMSCs secretion of an anti-inflammatory protein, TNF-α-stimulated gene/protein 6 (TSG-6). Wu et al. (2015) showed that *in vitro* assay, the MSC-CM decreased viability, α-SMA expression, and collagen secretion of human keloid fibroblasts. Besides, in a mouse dermal fibrosis model, MSC-CM infusion induced a significant decrease in skin fibrosis due to the TGF-β3 in CM-mediated therapeutic effects on preventing collagen accumulation (Wu et al., 2015). The application of ASCs and ASC-derivatives might also provide novel scarless repair methods. In the early stage of wound healing, exogenous ASC-exos promoted the expression of type I and type III collagen to shorten the healing time, and might inhibit collagen synthesis to minimize scar formation in the later period (Hu et al., 2016). This tendency followed the histological changes observed during the natural healing of soft tissue wounds. That is, collagen deposition was more important in the early phase of healing, while in the late phase of healing, matrix reconstruction was more critical. ASC-exos ameliorated ECM reconstruction and reduced the scar formation by regulating the ratios of type III collagen/type I collagen, TGF-β3/TGF-β1, and MMP-3/tissue inhibitor of metalloproteinases 1 (TIMP-1), as well as facilitating HDFs differentiation (Wang et al., 2017). The team of Wang et al. firstly developed the FHE hydrogel and FEP hydrogel scaffold both with stimuli-responsive ASC-exos. The ASC-exos released by these two carrier materials significantly increased the regeneration of skin appendages and reduced the formation of scar tissue (Wang C. et al., 2019) (Wang M. et al., 2019). The sustained release of bioactive exosomes helps to achieve better wound healing and scar removal.

## ASC-exos in Skin Flaps Injury

Flap transplantation is an essential method to repair refractory trauma and organ reconstruction, including the alar rim, external ear, and fingertip defects. The insufficient neovascularization and ischemia-reperfusion (I/R) injury are responsible for poor flap healing outcomes (Sorkin et al., 2020). ASC-based therapy has become an applicable method to prevent I/R injury for assisting flap transplantation. For instance, ASCs are able to enhance angiogenesis to increase the viability of chondrocutaneous composite grafts, for the application of defects in the nose, ear scales, and skin (Yucel et al., 2016).

Recent studies have shown that ASC-exos were hopeful to improve the survival status of skin flaps. By bioinformatics analysis, Xiong et al. pointed out that the miRNA-760 upregulation and miRNA-423-3p downregulation in ASC-exos could regulate the expression of ITGA5 and HDAC5 genes, respectively, consequently promoted the vascularization of the skin flap. In a leg wound model of rat, the microvascular angiography of 28 days post-flap transplantation revealed that the ASC-exos treated groups exhibited better vascularization degrees of the artificial dermis prefabricated flaps over control groups (Xiong et al., 2020). Undeniably, many differentially expressed miRNAs in ASC-exos are associated with the vascularization of flaps. The study by Pu et al. (2017) showed that IL-6-rich human ASC-exos promoted flap angiogenesis and flap repair after I/R injury in mice. In this case, the employment of ASC-exos to deliver IL-6 is beneficial for patient safety because it does not require the use of viral vectors. Bai et al. (2018) also showed that low-dose H_2_O_2_-stimulated ASC-exos could increase the neovascularization of the flap and relieve the inflammation and apoptosis after I/R injury, thus increasing the survival rate of the flap *in vivo*. In summary, ASC-exos play an important role in the vascularization of skin flaps, and thereby resolve the problem of insufficient neovascularization of the flaps, thus expanding the application of flap transplantation (Figure 1D).

## ASC-exos in Bone Tissue Damage

Fractures, tumor bone surgery, deformity, revision of the prosthesis, and osteomyelitis can be fully identified as segmental loss of bone structure. Particularly, bone regeneration is the main emphasis involving both surgery and aesthetics in craniofacial surgery. Exosomes, as nanoscale extracellular vesicles with an intercellular communication function, provide an excellent medium for the packaging and transportation of RNAs and proteins, benefiting for broad application in bone tissue engineering (Paduano et al., 2017). Besides, ASC-exos, with significant osteogenic induction ability, can effectively regulate the microenvironment of bone tissue by transporting a variety of bioactive molecules.

**FIGURE 2:**
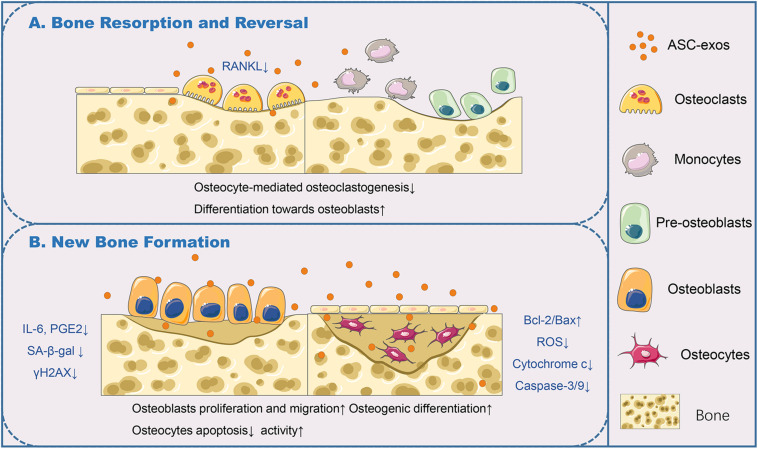
ASC-exos function in the bone remodeling cycle. **(A)** In bone resorption and reversal, ASC-exos could decrease the expression of RANKL, which was highly expressed by osteoclasts or their precursors for osteoclast activation, to antagonize osteocyte-mediated osteoclastogenesis. **(B)** In bone formation, ASC-exos possessed the ability of lowering the production of IL-6 and PGE2, downregulating SA-β-gal activity and reducing the accumulation of γH2AX in osteoblasts. Additionally, ASC-exos could upregulate the radio of Bcl-2/Bax, diminish the production of ROS and cytochrome c, and subsequent activation of caspase-3/9 in osteocytes. ASCs, Adipose-derived stem cells; ASC-exos, ASC-derived exosomes; RANKL, Receptor Activator of Nuclear Factor Kappa B Ligand; IL-6, Interleukin 6; PGE2, Prostaglandin E2; SA-β-gal, Senescence-Associated β-galactosidase; γH2AX, Phosphorylated Histone H2AX; Bcl-2, B-cell lymphoma/leukemia 2; Bax, Bcl-2-associated X protein; Caspase-3/9, Aspartate Proteolytic Enzyme 3/9.

The bone remodeling cycle is composed of consequential phases: resorption, reversal, and formation. Studies have found that osteocytes in bone tissues equip with many functions of coordinating the bone remodeling of osteoclasts and osteoblasts, which maintain the bone homeostasis (Borciani et al., 2020). An *in vitro* study confirmed that ASC-exos could antagonize osteocyte apoptosis triggered by ischemia and hypoxia, and decrease osteocyte-mediated osteoclastogenesis, which was attributed to the decrease in receptor activator of nuclear factor kappa b ligand (RANKL) expression (Ren et al., 2019). RANKL interacts with its receptor RANK, which is highly expressed by osteoclasts or their precursors and is essential for osteoclast activation (Figure 2A). ASC-exos can be used in tissue engineering combined with efficiently biocompatible efficient carriers to improve osteogenesis efficiency. According to a recent study, ASC-exos could be immobilized on the polydopamine-coating PLGA scaffolds. This cell-free nano-sized carrier enhanced bone regeneration significantly, at least partially through its osteoinductive effects and capacities of promoting MSCs migration and homing in the newly formed bone tissue (Li W. et al., 2018). It was definitely established that an ideal scaffold for exosomes loading would be biocompatible, biodegradable, and capable of controlled releasing exosomes. Chen S. et al. (2019) showed that the exosomes derived from miR-375-overexpressing ASCs incorporated with hydrogel possessed the ability to enhance bone regeneration in a rat model of calvarial defect. More effective and convenient loading strategies should be developed. Furthermore, appropriate changes in the culture conditions of ASCs will facilitate the production of customized ASC-exos. By using hypoxia/serum deprivation (H/SD) induced osteocyte apoptosis model with murine long bone osteocyte Y4 (MLO-Y4), Zhu et al. (2017) demonstrated ASC-exos could efficiently antagonize osteocyte apoptosis and osteocyte-mediated osteoclastogenesis, via upregulated radio of B-cell lymphoma 2 (Bcl-2)/Bcl-2-associated X protein (Bax), diminished production of ROS and cytochrome c, and subsequent activation of aspartate proteolytic enzyme 9 (caspase-9) and caspase-3. This result also provided the *in vitro* evidence of ASC-exos application in age-related bone disease. Lu et al. (2017) demonstrated that ASC-exos, especially primed by TNF-α pre-conditioned ASCs, could promote the proliferation and differentiation of human osteoblasts through Wnt signaling pathway. Therefore, the methods for producing specific ASC-exos, offer a promising approach to replace direct stem cell transplantation, further widening the application of exosomes in bone regeneration (Figure 2B).

Osteoarthritis (OA) is a common degenerative joint disease characterized by cartilage degeneration, synovitis, subchondral bone sclerosis, and osteophyte formation (Henrotin et al., 2012). Current treatments are basically symptomatic to handle pain and swelling, and mainly rely on antalgics and anti-inflammatory drugs. Articular cartilage has a limited potential to repair, with progressively more clinicians emphasizing cellular therapy. Multiple adipose tissue-associated components and extractions are promising to be applied in OA therapy, including SVF, ASCs, ASCs-exos, ASC-CM, and microfragmented adipose tissue (MFAT). In a clinical trial, Spasovski pointed out that the OA patients treated with single injection of ASCs showed significant cartilage restoration (Spasovski et al., 2018). Similarly, in another trial of OA patients received with ASCs, the ASCs might cause an immediate local response due to released paracrine factors and cytokines for OA amelioration (Pers et al., 2018). Tran et al. (2019) employed SVF to regenerate damaged knee cartilage of OA patients, revealing a trend toward a better efficacy of SVF with the microfracture method for OA treatment over a period of two years. Hong Z. et al. (2019) also suggested that intra-articular SVF injection was a safe treatment of OA, and could effectively relieve pain, improve function, and repair cartilage defects in patients with bilateral symptomatic knee osteoarthritis. MFAT could reduce the phase of cell manipulation without expansion or enzymatic treatment in a short period (Paolella et al., 2019). In an inflammatory cell model of OA synoviocytes, MFAT reduced typical macrophages markers and its potentiality to induce an anti−inflammatory effect to address OA (Mautner et al., 2019).

As the cytoprotective and anti-inflammatory properties of ASCs in human chondrocytes and experimental OA may be mediated by paracrine effects, the paracrine mediators ASC-exos are attractive for alternative therapies of OA. ASC-exos might be safer, cheaper, and more effective OA therapy (Pers et al., 2015). ASC-exos are able to downregulate inflammation and oxidative stress, which might successfully mediate antisenescence in OA (Wang Q. et al., 2020). The intra-articular injection of ASC-exos could inhibit cartilage and subchondral bone degradation, decrease osteophyte formation, and anti-synovial inflammation, thus slowing the progression of OA (Zhang R. et al., 2019). Tofiño-Vian et al. (2017) showed that both ASC-CM and ASC-exos lowered the production of IL-1β-stimulated inflammatory mediators IL-6 and prostaglandin E2 (PGE2), down-regulated senescence-associated β-galactosidase (SA-β-gal) activity, and reduced the accumulation of phosphorylated histone H2AX (γH2AX), in OA osteoblasts. Furthermore, in the next study, they confirmed that microvesicles and exosomes secreted from ASCs could affect the metabolism of OA chondrocytes by modulating inflammatory and degradative pathways associated with joint destruction (Tofiño-Vian et al., 2018). Zhao et al. (2020) separated the patient ASC-exos, which exerted a strong stimulatory effect on chondrocyte migration and proliferation with the upregulation of miR-145 and miR-221 in the model of the inflammation-inflicted oxidative stress. Woo et al. also confirmed that human ASC-EVs could potentially protect cartilage from degeneration and could delay cartilage degeneration in OA rat and mouse models. The mechanism was probably that human ASC-EVs suppressed IL-1β up-regulated catabolic molecules and enhanced type II collagen expression in human OA chondrocyte (Woo et al., 2020). Ragni established an *in vitro* model of human fibroblast-like synoviocytes (FLSs) from OA patients, showing that ASC-EVs possessed the immunoregulatory properties for OA regulation and that hyaluronan was involved in ASC-EVs internalization in FLSs (Ragni et al., 2019).

These pioneering results reinforced the great prospects for ASC-exos and ASC-EVs as a novel therapeutic option for OA. However, the number of studies is small. In the context of OA, although the ASCs and SVF have been confirmed their clinically therapeutic efficacy and safety, the ASC-exos therapies have not yet been used in clinical trials. There still needs to execute a detailed exploration in large cohorts to investigate that the functions and mechanisms of ASC-exos are necessary. In pre-clinical studies, the optimized conditions and obtainments for ASC-exos *in vitro* and the mechanisms of ASC-exos *in vivo* require further studies. In clinical trials, the ASC-exos based therapy should set optimal criteria, including exosome concentration and dose, injection times and intervals. In addition, the comprehensive immune impact following the ASC-exos administration should be performed to determine the immune response of the recipient. Totally, ASC-exos may represent the effective clinical strategy of OA once trials have been fully controlled and their benefits and safety have been fully assessed.

## ASC-exos in Obesity

**FIGURE 3:**
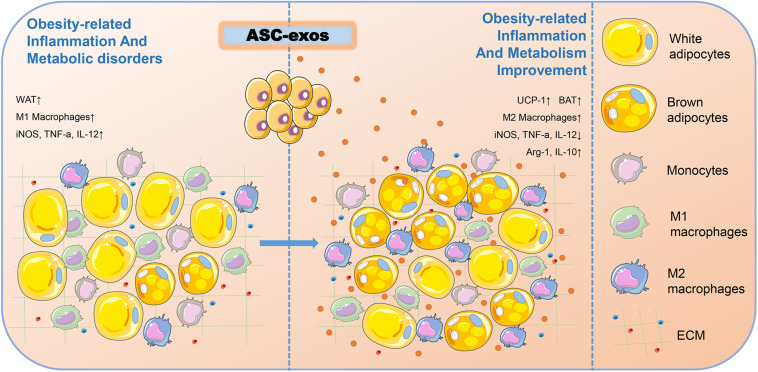
ASC-exos could improve obesity-related inflammation and metabolism. In the obesity microenvironment of mice, ASC-exos was able to reduce the secretion of iNOS, TNF-a, and IL-12, and increase the secretion of Arg-1 and IL-10 to activate M2 macrophage polarization, thus ameliorating WAT inflammation. ASC-exos also increased the expression of UCP-1 to promote WAT beiging, thereby improving obesity-related inflammation and metabolism. ASCs, Adipose-derived stem cells; ASC-exos, ASC-derived exosomes; WAT, White Adipose Tissue; BAT, Brown Adipose Tissue; iNOS, Inducible Nitric Oxide Synthase; TNF-α, Tumor Necrosis Factor Alpha; IL-12, Interleukin 12; Arg-1, Arginase 1; IL-10, Interleukin 10; UCP-1, Uncoupling Protein 1.

Obesity is a growing health pandemic whose global prevalence has increased dramatically over the last few decades. In addition to bringing the physical changes, obesity also causes considerable obesity-related inflammation and metabolic disorders, including dysfunction of adipose tissue and insulin resistance in key metabolic organs and insufficient secretion of insulin by the pancreas (Zhang B. et al., 2019). Apart from the energy storage function, the adipose tissue is also an important endocrine tissue and a great source of ASC-exos. ASCs play critical roles in controlling obesity-associated inflammation and metabolic disorders. Thus, the secretion quantity and function of ASC-exos are hypothetically shaped by obesity. Zhao et al. (2018) found that ASC-exos could transfer into macrophages to induce anti-inflammatory M2 phenotypes through the transactivation of arginase 1 (Arg-1) and IL-10 by exosome-carried active STAT3, and increase the expression of uncoupling protein 1 (UCP-1) to promote white adipose tissue (WAT) beiging, thereby improving obesity-related inflammation and metabolism (Figure 3). Mechanistically, this study delineated a novel exosome-mediated ASC-macrophage cross-talk that facilitated immune and metabolic homeostasis in WAT, thus providing a potential therapy for obesity and diabetes.

Extracellular vesicles belong to a heterogeneity system, including exosomes, apoptotic bodies, microvesicles (Perez-Hernandez et al., 2017). It is worth noticing on ASC-EVs peculiarity in different physiological or pathological contexts. The concrete use of ASC-EVs in cell-based therapy in the obese setting should be taken into account. Some studies suggested that obesity or diabetes could impair the capacity of ASCs for anti-inflammation and wound healing, as well as influence the production and bioactivities of ASCs-exos, thus increasing the risk for immune or metabolic disorders (Strong et al., 2016). Togliatto et al. (2016) investigated the therapeutic impact of ASC-EVs recovered from obese subjects visceral and subcutaneous tissues. Compared with ASC-EVs from non-obese subjects, ASC-EVs from obese subjects showed impaired angiogenic potential *in vitro* because of the decrease of EVs cargoes including VEGF, MMP-2, and miRNA-126 (Togliatto et al., 2016). Obesity impacts on ASC-EVs and ASC-exos pro-angiogenic potential and may raise more concerns about these crucial tissue repair mediators.

## ASC-exos in Fat Grafting

Fat grafting has been gaining large attention in tissue augmentation over the past decades for hemifacial atrophy, lipodystrophy, and breast reconstruction (Tan and Loh, 2017). Both ASCs and fat graft can exert a wrinkle-reducing effect and synergistically affect collagen synthesis and neovascularization (Kim et al., 2019). However, the survival rate of fat grafts remains unsatisfied due to the devascularization and ischemic injury of adipose tissues made by liposuction, injection, and long-term fat absorption. Several technical modifications have been described to enhance fat graft survival with more complete adipose tissue structure. Cell-assisted lipotransfer (CAL) is an efficient technique that mixes ASCs-rich SVF with lipoaspirate, for reinforcing adipogenesis and angiogenesis to augment fat graft reliability (Yoshimura et al., 2008). Importantly, Kølle et al. (2020) conducted a randomized controlled clinical trial comparing fat grafts enriched with *ex vivo*-expanded autologous ASCs to non-enriched fat grafts in breast augmentation, demonstrating that ASCs significantly improved the volume retention of fat grafts compared with conventional fat grafting and no adverse effects were observed. This result further confirmed the significance of CAL in both reconstructive and cosmetic volume restoration. However, limitations of cell-based therapies have constrained their use, including uncommitted differentiation, unwanted side effects, immune rejection, and regulatory hurdles (O’Halloran et al., 2018). ASC-exos have been identified to motivate functional recovery in fat grafting and filling.

**FIGURE 4:**
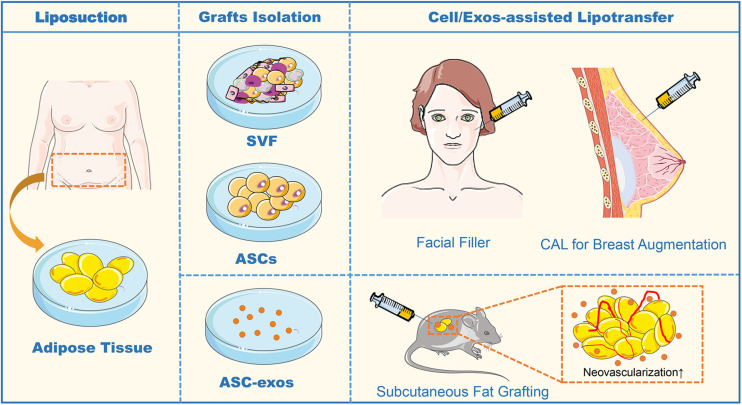
The sequence flow diagram of fat grafting. In the clinical application of fat grafting for facial filler and breast augmentation, sterile adipose tissue is collected through liposuction. After enzyme digestion and centrifugation of the collected adipose tissue, the obtained heterogeneous mixture of endothelial cells, smooth muscle cells, fibroblast, pericytes, mast cells, and preadipocytes is named SVF. In CAL, half the volume of the aspirated fat is processed for isolation of the SVF containing ASCs, and the other half of the aspirated fat is prepared for grafting. Finally, the SVF-supplemented fat is injected into the target sites of grafting. In animal studies of the subcutaneous fat grafting, co-transplantation of ASC-exos with adipocytes can effectively promote the neovascularization to enhance survival in the fat grafts. ASCs, Adipose-derived stem cells; ASC-exos, ASC-derived exosomes; SVF, Stromal Vascular Fraction; CAL, Cell-Assisted Lipotransfer, CAL.

Recently, Han et al. (2018) discovered that co-transplantation of ASC-exos and hypoxia-treated ASC-exos in nude mouse models of subcutaneous fat grafting both could participate in neovascularization and attenuate inflammation in the grafts. In the subsequent study, they further investigated the molecular mechanism of hypoxia-enhancing promoting the effect of ASC-exos in fat grafting, raising that the hypoxia-treated ASC-exos significantly enhance the angiogenesis of the ischemic adipose tissue by regulating VEGF/VEGF-R signaling pathway (Han et al., 2019). As the functional nanovesicles secreted by ASCs, ASC-exos possess more advantages in improving the volume retention rate of fat grafts than ASCs. Chen B. et al. (2019) have shown that ASCs-exos were equivalent to ASCs in improving the survival of fat grafts by up-regulating early inflammation and enhancing angiogenesis in mice. Whereas during the mid to late stages of fat grafting, ASC-exos exerted a pro-adipogenic effect and also increased collagen synthesis level, similarly, to their source of ASCs (Chen B. et al., 2019). Zhu et al. (2020) found that in an *in vivo* mouse model of autologous fat grafting, grafts treated by ASC-EVs significantly exhibited survival with more neovascularization, increased fat retention, and decreased fibrosis and necrosis. The ASC-EVs uptake by macrophages promoted M2 type polarization and catecholamine secretion, thus the M2 macrophages-CM could enhance browning adipose differentiation with enhanced energy expenditure (Zhu et al., 2020). These results suggest that, as a cell-free strategy, ASC-exos could be an effective and appealing path to heighten graft survival in lipotransfer (Figure 4). However, a major drawback of fat grafting is the unpredictability of the clinical outcome since high volume absorption rates are common. It is not yet clear how long this effect lasts or whether ASC-exos from both healthy individuals and diseased patients are equally effective. Further experimental and clinical studies are required to determine the optimal concentration and source of ASC-exos enrichment required to improve fat graft without side effects.

## ASC-exos in Breast Cancer and Breast Reconstruction

Adipocytes are the abundant cellular components in the breast cancer microenvironment. The invasion of breast cancer cells leads to the proximity of cancer cells and adipocytes, which have been referred to as cancer-associated adipocytes (CAAs) (Choi et al., 2018; De Lope et al., 2018). CAAs exhibit a fibroblastic-like morphology and overexpressed ECM proteins. CAAs can interact with cancer cells via several ways to trigger breast cancer initiation, metastasis, angiogenesis, and cachexia. Studies have demonstrated the survival rate, growth, and invasiveness of tumors after interacting with ASCs, which are CAAs components. By excreting a variety of bioactive factors including visfatin, adipsin, CCL5, IGF, HGF, VEGF, IL-8, and TGF-β, ASCs can exert biological influences on proliferation, epithelial-mesenchymal transition, and metastasis (Huang et al., 2019; Goto et al., 2019). ASCs could differentiate into cancer-associated fibroblasts in breast cancer environment, especially with a more pronounced effect on obese patients (Cho et al., 2012; Ecker et al., 2019).

The exosomes, released from the adipocytes and ASCs, are emerging as a new type in the crosstalk between breast tumors and adipocytes (Sauter et al., 2019; Teufelsbauer et al., 2019). Previously, Lin et al. (2013) investigated the effect of ASC-exos on breast cancer MCF7 cells, showing that ASC-exos activated the Wnt and Hh signaling pathways to strengthen tumor cell migration. On the other hand, the breast cancer-derived exosomes could reciprocally shape the function of adipocytes. A recent study confirmed that miRNA-144 and miRNA-126-secreting exosomes from breast cancer cells co-cultured with adipocytes could promote cancer metastasis by inducing beige/brown differentiation and reprogramming the metabolism in surrounding adipocytes (Wu et al., 2019). Interestingly, Lee et al. (2019) emphasized the effect of cancer stem cells (CSCs) treated with exosomes derived from osteogenic differentiating human ASCs. The results showed that the expression of drug-resistance genes (ATP binding cassette transporter), the breast cancer gene family (BCRA1 and BCRA2), and the ErbB gene family were all significantly decreased in CSCs (Lee et al., 2019). The osteoinductive ASCs-exos could be a biochemical cue for CSCs reprogramming into non-tumorigenic cells and contribute to overcoming therapeutic resistance. He et al. (2018) noted that MSC-CM could also suppress the growth of breast cancer cells by inhibiting the STAT3 signaling pathway and MSC-CM combined with radiotherapy significantly delayed xenograft tumor growth in mice.

For patients with breast cancer, breast reconstruction requires the unification of tumor safety and aesthetics. Fat grafting using mammaplasty might complicate breast imaging and breast cancer surveillance due to the varying levels of nodule formation and calcifications in breast tissue (Bayram et al., 2019). The ASCs assisted fat transplantation is a common technique for CAL which makes great improvements in the survival rate of transplanted fat with less fat resorption and necrosis for the favorable aesthetic outcome of breast augmentation. Nevertheless, tumor safety in breast reconstruction is the primary consideration (O’Halloran et al., 2017). Silva et al. (2019) suggested that fat transplantation did not promote tumor proliferation and metastasis in mouse models of residual breast cancer. Krastev et al. carried out a long-term follow-up trail of 587 breast cancer patients who underwent autologous fat transplantation and traditional breast reconstruction, respectively. The results showed that after 5 years the local recurrence rate of breast cancer had no significant difference in these two methods (Krastev et al., 2019). Similarly, Gentile et al. (2019a) also conducted a 3-year follow-up of 121 patients with breast cancer who underwent adipose stromal cell-enhanced engineering fat transplantation, which also showed satisfactory safety and effectiveness. Thus, these clinical studies using ASCs report safety data in breast reconstruction.

To some extent, exosomes originate from the tumor-adipocytes interaction in a complex metabolic network favoring malignant progression. However, at this stage, there are limited studies on ASC-exos used for breast reconstruction. In clinical trials and basic experiments, it is worth noting that the role of ASC-exos in breast reconstruction after breast cancer surgery is not entirely clear. To avoid the tumorigenic potential risk, the mechanisms of ASCs and ASCs-exos in breast cancer and breast construction remain to be carefully elucidated. Additionally, it is important to conduct randomized trials for illuminating the safety and efficacy of transplanted ASCs or ASC-exos, in comparison to commonly applying with conventional techniques.

## Conclusion and Perspective

Adipose-derived stem cells are stem cell populations within the adipose stromal compartment that have multiple differentiation potentials, easy acquisition, and the high yield, making ASCs attractive for tissue engineering and cell therapy as an ideal stem cell source. ASC-exos, containing important paracrine mediators, have received much attention recently for functioning in intercellular communication. As cell-to-cell messengers, ASC-exos are valuable supplement with the regenerative and reconstructive strategies, and is particularly successful and safely applied to chronic wounds, scars, bone injuries, and cosmetic surgery. Both ASCs and ASC-exos possess huge application potential on the tissue regenerative field in plastic and cosmetic surgery.

In terms of source and production, ASCs boast the benefits that the stem cell yield from fat is much greater than that obtained from other sources. ASC-exos possess advantages of huge sources and high availability, indicating that ASC-exos can be an alternative when exosomes from other sources have difficulties to extract or are not suitable for therapy (Ha et al., 2020). ASC-exos and other MSC-exos have similar characteristics, such as morphology and cell surface markers, but some important biological differences have been found in proliferation, gene expression, differentiation ability, and immunosuppressive pathways. For exosomes from different sources of MSCs, they partly contain similar proteins. Interestingly, Villatoro et al. (2019) showed that canine ASCs had advantages of proliferative capacity, whereas canine BMSCs showed a significantly higher secretory production of some soluble factors. Therefore, when selecting the source of MSCs, those biological differences should be considered for cell implantation or the secretome is directly used for specific clinical applications. In addition, it is obvious that those of MSC-exos vary according to the origin of MSCs. However, comparative studies of MSC-exos by their tissue origin are still limited, and only a few reports have compared different exosomes within the same study. For example, compared with human BMSC-exos, ASC-exos exhibited a higher activity of neprilysin, which was an amyloid β peptide degrading enzyme, suggesting the therapeutic relevance of ASC-exos in Alzheimer disease (Katsuda et al., 2013). ASCs might be safer and more effective than that from BMSCs, including lack of major histocompatibility complex (MHC) class II on ASCs, induction of higher levels of anti-inflammatory macrophages and pro-lymphangiogenic activity (Maguire, 2019).

Furthermore, compared with ASCs, ASC-exos offer distinct advantages that uniquely position them as highly effective bioactive constituents. The proposed several main reasons are as follows based on previous reports: (1) Biosafety: exosomes, including ASCs-exos, are naturally occurring secreted membrane vesicles from the releasing cells with lower immunogenicity, posing the favorable biosafety of ASC-exos. (2) Biological activity: although without similar self-differentiation function as ASCs, ASCs-exos contain a broad repertoire of cargoes, including nucleic acid, proteins, and enzymes for modulating multiple cellular activities, acting in both immediate and remote areas in a paracrine manner. (3) Stability: the ASC-exos are comprised of naturally bimolecular phospholipid structure, providing sufficient stability to avoid biodegradation. Thus, ASC-exos are very well tolerated in biological fluids along with the ubiquitous presence. (4) Carrier features: ASCs-exos function as a carrier for itself, also can be used as a component uploaded in well-designed biomedical materials. Due to the intrinsic homing capacity or artificially modified targeting ability, ASC-exos can serve as stable and effective carriers to load specific proteins, lipids, and genetic material, and preferentially transport it to targeted tissues or organs. Exosome-based delivery systems may be of precedence in the treatment of diseases attributing to their endogenous origin, which minimizes the immunogenicity and toxicity and exerts the optimal effect. The development of multifunctional bioactive biomaterials with long-term ASC-exosomes release is also very important to synergistically enhance tissue regeneration and therapy. With the above desirable properties, ASC-exos hold clinically promising potential in the novel cell-free therapeutic strategies. Therefore, as mentioned in this review, ASC-exos are expectantly recognized as new candidates for the skin anti-aging therapy, skin inflammation treatment, wound and scar repair, flap grafting, bone tissue repair, obesity prevention, fat transplantation, breast cancer, and breast reconstruction (Table 2). Collectively, these findings reinforce the significance of ASC-exos-participated cell communication and applications in plastic and cosmetic surgery. Though lacking adequate application in clinical practice, ASC-exos are playing an increasingly greater role especially in maximizing the therapeutic effect of dermopathic features and tissue reconstruction.

**TABLE 2:** The mechanisms and functions of ASC-exos in tissue regeneration.

Disease	Source	Model	Function	Mechanism	References
Skin aging	Human ASC-CM	Photoaging-induced HDFs and HaCaTs	Photoaging prevention	Downregulate the activation and transcription of UVB-induced signaling pathways and upregulate the expression of antioxidant response elements	Li et al., 2019
	Human ASC-CM	Photoaging-induced HDFs	Photoaging prevention	PDGF-AA in ASC-CM promoted HDFs proliferation and activated PI3K/Akt signal pathway to facilitate ECM deposition and remodeling	Guo et al., 2020
	Human BMSC-exos	Photoaging-induced HDFs and mice	Photoaging prevention	Produce ROS at a low level, downregulate TNF-α, upregulate TGF-β to increase MMP-1 and procollagen type I expression for collagen synthesis	Hu et al., 2019
Atopic dermatitis	Human ASC-exos	AD model of NC/NGA mice	Dermatitis improvement	Decrease the levels of inflammatory cytokines and reduce the number of eosinophils in the blood, and the infiltration of mast cells, dendritic epidermal cells	Cho et al., 2018
	Human ASC-exos	AD model of SKH-1 mice	Epidermal Barrier Repair	Reduce trans-epidermal water loss and enhance epidermal lamellar bodies and form lamellar layer at the interface of the SC and stratum granulosum.	Shin et al., 2020
Skin wound	Human ASC-exos	Skin lesion model of HaCaTs	HaCaTs viability enhancement	Foster HaCaTs proliferation, migration, and inhibit apoptosis through Wnt/β-catenin signaling pathway	Ma et al., 2019
	Human ASC-exos	Skin lesion model of HaCaTs and HDFs	HaCaTs and HDFs viability enhancement	ASC-exos containing MALAT1 could mediate H2O2-induced wound healing via targeting miR-124 through activating the Wnt/β-catenin pathway	He et al., 2020
	Human ASC-exos	full-thickness skin wound of mice	Wound healing	Promote fibroblasts proliferation and migration and optimize collagen deposition via the PI3K/Akt signaling pathway to accelerate wound healing.	Zhang et al., 2018
	Human ASC-exos	Diabetic foot ulcer of rat	Wound healing	ASC-exos overexpressing-Nrf2 promoted the proliferation and angiogenesis of endothelial cells, and increased the expression of wound growth factor, decreased the levels of inflammation and oxidative stress-related proteins.	Li X. et al., 2018
	Human ASC-exos	Full layer skin wound of mice	Wound healing	ASC-exos overexpressing miRNA-21 enhanced the migration and proliferation of the HaCaTs by increasing the MMP-9 expression through the PI3K/AKT pathway	Yang et al., 2020
	ASC-exos	Skin wound of diabetic mice	Wound healing	mmu_circ_0000250 enhanced the therapeutic effect of ASCs-exosomes to promote wound healing in diabetes by absorption of miR-128-3p and upregulation of SIRT1	Shi et al., 2020
Scar formation	Human ASC-exos	Skin wound of mice	Scar removal	Inhibit collagen expression to reduce scar formation in the late stage of wound healing	Hu et al., 2016
	Human ASC-exos	Skin wound of mice	Scar removal	Regulate the ratios of type III collagen/type I collagen, TGF-β3/TGF-β1, and MMP-3/TIMP-1, as well as facilitating HDFs differentiation	Wang et al., 2017
Skin flap injury	Human ASC-exos	Artificial dermis prefabricated flap and leg wound of rat	Flap vascularization	Upregulation of miRNA-760 and downregulation of miRNA-423-3p in ASC-exos could regulate the expression of ITGA5 and HDAC5 genes, respectively, to promote the vascularization of the skin flap	Xiong et al., 2020
Skin flap I/R injury	Human ASC-exos	Skin flap I/R injury of mice	Flap repair	IL-6 highly contained in ASC-exos could enhance skin flap recovery and angiogenesis after I/R injury	Pu et al., 2017
	Human ASC-exos	Skin flap I/R injury of mice	Flap repair	H_2_O_2_-treated ASC-exos increased the neovascularization and relieve the inflammation and apoptosis of the flap after I/R injury	Bai et al., 2018
Bone defect	Human ASC-exos	Hypoxic-ischemic osteocyte	Osteogenesis	Ameliorate osteocyte apoptosis and osteocyte-mediated osteoclastogenesis by lowering the expression of RANKL	Ren et al., 2019
	Human ASC-exos	Calvarial defects of rats	Bone formation	ASC-exos overexpressing miRNA-375 were absorbed by hBMSCs, and inhibit the expression of IGFBP3 to exert osteogenic effects	Chen S. et al., 2019
	Human ASC-exos	Human primary osteoblastic cells	Bone formation	TNF-α-preconditioned ASC-exos promoted the proliferation and differentiation of human osteoblasts through the Wnt signaling pathway	Lu et al., 2017
Osteoarthritis	Human ASC-exos	OA model of osteoblasts	Inflammation improvement	Downregulate SA-β-gal activity and the accumulation of γH2AX	Tofiño-Vian et al., 2017
	Human ASC-exos	Chondrocytes stimulated with H_2_O_2_	Chondrogenesis	Downregulated the pro-inflammatory markers IL-6, NF-κB and TNF-α, while they upregulated the anti-inflammatory cytokine IL-10 when co-cultured with activated synovial fibroblasts, promoted chondrogenesis in periosteal cells and increased collagen type II and β-catenin	Zhao et al., 2020
Obesity	Mouse ASC-exos	Obese mice	Obesity prevention	Activate M2-type macrophage polarization, improve inflammation, and promote the browning of white adipose tissue	Zhao et al., 2018
Fat grafting	Human ASC-exos	Mice	Fat grafts survival promotion	Hypoxia-treated ASC-exos enhanced the angiogenesis by regulating the VEGF/VEGF-R signaling pathway	Han et al., 2019
	Mouse ASC-exos	Mice	Fat grafts survival promotion	Promote angiogenesis and up-regulate early inflammation, exert proadipogenic effect and increase collagen synthesis during the mid to late stages	Chen S. et al., 2019
Breast cancer	Human ASC-exos	Breast cancer MCF-7 cells	Tumor progression	Activate the Wnt and Hh signaling pathways to strengthen the growth of MCF-7 cells	Lin et al., 2013

*ASCs, Adipose-derived stem cells; ASC-exos, ASC-derived exosomes; HDFs, Human Dermal Fibroblasts; HaCaTs, Human Keratinocytes; UVB, Ultraviolet B; ASC-CM, ASC-Conditioned Medium; ECM, Extracellular Matrix; H_2_O_2_, Hydrogen Peroxide; PDGF-AA, Platelet-Derived Growth Factor-AA; ROS, Reactive Oxygen Species; MMP-1/9, Matrix Metalloproteinase 1/9; TNF-α, Tumor Necrosis Factor Alpha; TGF-β, Transforming Growth Factor Beta; IL-4/5/6/13, Interleukin 4/5/6/13; VEGF, Vascular Endothelial Growth Factor; Nrf2, NF-E2-related factor 2; TIMP-1, Tissue Inhibitor of Metalloproteinases-1; I/R, Ischemia-Reperfusion; RANKL, Receptor Activator of Nuclear Factor Kappa B Ligand; SA-β-gal, Senescence-Associated β-galactosidase; γH2AX, Phosphorylated Histone H2AX; Bcl-2, B-cell lymphoma/leukemia 2; Bax, Bcl-2-associated X protein.*

Nevertheless, there are still some challenges in the development of ASC-exos application. Firstly, obtaining ASCs continues to be an inconvenience. The sources of ASCs, as well as the separation and cultivation methods, medium composition and dosage, cell passage, cell fusion and viability, mycoplasma, and other microbial contamination, all should be tightly controlled to maintain reliable biological efficacy and ASC-exos with high quality. Secondly, the extracted ASC-exos might have low purity and yield in the lab. Nowadays ASC-exos separation methods include ultracentrifugation, exclusion, ultrafiltration, two-aqueous system, immunoaffinity, and polymer precipitation. Although the ultracentrifugation is the most common method for largely separating exosome, but accompanied by the shortcomings of time-consuming, labor-intensive, costly instrumentation, and multiple overnight centrifugation steps. As it is overly idealistic to completely isolate ASC-exos from other components, the efficient, appropriate, and affordable techniques should be thoughtful to acquire exosomes. These technologies still need to achieve equilibrium in improving the yield and purity of ASC-exos.

Thirdly, given that plastic surgery frequently performs liposuction and autologous fat transplantation, plastic surgeons and researchers have unique advantages in obtaining ASCs and ASCs-exos. However, in the application of ASC-exos in tissue regeneration of plastic and cosmetic surgery, there are many aspects worth noting. Emerging studies have reported that ASC-exos can be utilized in plastic and cosmetic surgery, but almost all these studies are limited in cellular and animal assays, without the large scale exploration of clinical trials like ASCs. In addition, exosomes could be absorbed on human skin, but even if in the existing studies, there is still lack of enough reports in skin aging, atopic dermatitis and skin flaps injury, which are important and intractable skin disease. Therefore, the actual clinical prospects of ASC-exos are almost a blank, also meaning that there exist vast space for development. Another important point is that auxiliary therapy of ASC-exos. Clinically, for plastic and cosmetic surgery-related diseases, laser therapy, drug therapy, tissue filling and surgical operation are common treatments and achieve good clinical effects. Nevertheless, to a certain extent, ASC-exos can only be used as a supplementary treatment, rather than a sole therapy in the beginning. Most of the existing studies focus on ASC-exos as the main or sole treatment. Hence, whether ASC-exos possess synergistic effects or inhibitory effects on the above-mentioned common treatments needs further research.

According to the above considerable insights into the ASC-exos applications and limitations, it needs to carry out more comprehensive researches in the following aspects. (1) Quality control. There exists a substantial degree of heterogeneity in dosing regimens in the reported cases. For better outcomes of plastic and cosmetic surgery, ASC-exos utilization details, including the storage conditions, effective doses, concentration, and period of treatment, are all the important points. It is necessary to further explore suitable microenvironmental conditions or genetic engineering techniques to ascertain the efficiency of ASC-exos treatment. (2) Components and functions. The ASC-exos are comprised of multiple bioactive components. The complex multi-component substances in ASC-exos may produce diverse biological characteristics when finally used in practice. The propensity for some controversial effects of ASC-exos is contingent upon the type and state of the host cells, the type and state of the recipient cells, and the interacting microenvironments. For possibly utilizing ASC-exos in clinical application, a deep understanding of ASC-exos and their components is the priority. Identifying the key components and reforming ASC-exos to overexpress these components might maximize the therapeutic effect while reducing the side or off-target effects. In the subsequent studies, ongoing advances in the analysis of the function of ASC-exos will probably unravel the ASC-exos characteristics, allowing deepening the understanding of their role in pathogenesis and regeneration properties. (3) Carrier peculiarity exploration. ASC-exos is of carrier peculiarity due to the intricate structure of exosomes. It means that exosomes are the ideal therapeutic delivery system. ASC-exos are effective tools for cargo transportation of effective therapeutic agents with lower immunogenicity and toxicity. On the other hand, ASC-exos could also be uploaded in the specific nanomaterials or hydrogel materials to promote skin repair. Engineering ASC-exos to be effective and safe requires a comprehensive understanding of their necessary components, including but not limited to membrane stability, architecture, and packaging of the interior components. (4) Large clinical trials. At present, excavations on ASC-exos studies belong to basic research or animal level. These experiments can not clearly and actually reflect the ASC-exos usages and their physiological levels *in vivo*. In fat transplantation, it is of great value to clinically explore whether the exogenous ASC-exos could be used for cell transplantation with safety and effectiveness. Especially, clinical implementation of any operation must be based on safety. However, the roles of ASC-exos in some diseases remain controversial. The oncological safety of ASC-exos in breast cancer and breast reconstruction is worthy of extreme attention. Moreover, the ASC-exos impacts on the common treatment of plastic and cosmetic surgery are urgent clinical problems. In the end, larger prospective, blinded, randomized clinical trials are in urgent need to further establish the long-term effectiveness, safety and dose of ASC-exos in humans. Collectively, ASC-exos are promising candidates for cell-free therapy strategy and deserve intense investigation to accelerate ASC-exos applications in tissue regeneration of plastic and cosmetic surgery.

## Author Contributions

MX and QZ performed the literature search and wrote the manuscript. MW and YW conceived the project and revised the manuscript. WH, CZ, WL, and YY edited the manuscript. All authors reviewed the manuscript and approved the final version.

## Conflict of Interest

The authors declare that the research was conducted in the absence of any commercial or financial relationships that could be construed as a potential conflict of interest.

## Abbreviations

γ H2AX: Phosphorylated Histone H2AX
AKT: Protein Kinase B
AP-1: Activator Protein 1
Arg-1: Arginase 1
ASC-CM: ASC-Conditioned Medium
ASC-exos: ASC-derived exosomes
ASCs: Adipose-derived Stem Cells
BAT: Brown Adipose Tissue
Bax: Bcl-2-associated X Protein
Bcl-2: B-cell lymphoma/leukemia 2
CAL: Cell-Assisted Lipotransfer
Caspase-3/9: Aspartate Proteolytic Enzyme 3/9
ECM: Extracellular Matrix
EVs: Extracellular Vesicles
H_2_O_2_: Hydrogen Peroxide
HaCaTs: Human Keratinocytes
HDFs: Human Dermal Fibroblasts
I/R: Ischemia-Reperfusion
IFN- γ: Interferon Gamma
IL-4/5/6/13: Interleukin 4/5/6/13
iNOS: Inducible Nitric Oxide Synthase
MAPKs: Mitogen-Activated Protein Kinases
MMP-1/9: Matrix Metalloproteinase 1/9
MSCs: Mesenchymal Stem Cells
NF- κ B: Nuclear Factor Kappa B
NOX-1/4: NADPH Oxidase 1/4
Nrf2: NF-E2-related factor 2
PDGF-AA: Platelet-Derived Growth Factor-AA
PGE2: Prostaglandin E2
PI3K: Phosphatidylinositol-4,5-Bisphosphate 3-Kinase
RANKL: Receptor Activator of Nuclear Factor Kappa B Ligand
ROS: Reactive Oxygen Species
SA- β-gal: Senescence-Associated β-galactosidase
SMP30: Senescence Marker Protein 30
SVF: Stromal Vascular Fraction
TGF- β: Transforming Growth Factor Beta
TIMP-1: Tissue Inhibitor of Metalloproteinases 1
TNF- α: Tumor Necrosis Factor Alpha
TSG-6: TNF-Alpha-Stimulated Gene/Protein 6
UCP-1: Uncoupling Protein 1
UVB: Ultraviolet B
VECs: Vascular Endothelial Cells
VEGF: Vascular Endothelial Growth Factor
WAT: White Adipose Tissue.

## References

[B1] Alves N. O. da Silva G. T. Weber D. M. Luchese C. Wilhelm E. A. Fajardo A. R. (2016). Chitosan/poly(vinyl alcohol)/bovine bone powder biocomposites: a potential biomaterial for the treatment of atopic dermatitis-like skin lesions. *Carbohydr. Polym.* 148 115–124. 10.1016/j.carbpol.2016.04.049 27185122

[B2] Bai Y. Han Y. Yan X. Ren J. Zeng Q. Li X. (2018). Adipose mesenchymal stem cell-derived exosomes stimulated by hydrogen peroxide enhanced skin flap recovery in ischemia-reperfusion injury. *Biochem. Biophys. Res. Commun.* 500 310–317. 10.1016/j.bbrc.2018.04.065 29654765

[B3] Bajek A. Gurtowska N. Olkowska J. Kazmierski L. Maj M. Drewa T. (2016). Adipose-derived stem cells as a tool in cell-based therapies. *Arch. Immunol. Ther. Exp.* 64 443–454. 10.1007/s00005-016-0394-x 27178663 PMC5085986

[B4] Bayram Y. Sezgic M. Karakol P. Bozkurt M. Filinte G. T. (2019). The use of autologous fat grafts in breast surgery: a literature review. *Arch. Plast. Surg.* 46 498–510. 10.5999/aps.2019.00416 31775202 PMC6882697

[B5] Bellei B. Migliano E. Tedesco M. Caputo S. Papaccio F. Lopez G. (2018). Adipose tissue-derived extracellular fraction characterization: biological and clinical considerations in regenerative medicine. *Stem Cell Res. Ther.* 9:207. 10.1186/s13287-018-0956-4 30092820 PMC6085647

[B6] Bi H. Li H. Zhang C. Mao Y. Nie F. Xing Y. (2019). Stromal vascular fraction promotes migration of fibroblasts and angiogenesis through regulation of extracellular matrix in the skin wound healing process. *Stem Cell Res. Ther.* 10 1–21. 10.1186/s13287-019-1415-6 31623669 PMC6798485

[B7] Borciani G. Montalbano G. Baldini N. Cerqueni G. Vitale-Brovarone C. Ciapetti G. (2020). Co-culture systems of osteoblasts and osteoclasts: simulating in vitro bone remodeling in regenerative approaches. *Acta Biomater.* 108 22–45. 10.1016/j.actbio.2020.03.043 32251782

[B8] Bukowska J. Alarcon Uquillas A. Wu X. Frazier T. Walendzik K. Vanek M. (2020). Safety of human adipose stromal vascular fraction cells isolated with a closed system device in an immunocompetent murine pressure ulcer model. *Stem Cells Dev.* 29 452–461. 10.1089/scd.2019.0245 31992147 PMC7153633

[B9] Chang Y. W. Wu Y. C. Huang S. H. Wang H. M. D. Kuo Y. R. Lee S. S. (2018). Autologous and not allogeneic adipose-derived stem cells improve acute burn wound healing. *PLoS One* 13:e0197744. 10.1371/journal.pone.0197744 29787581 PMC5963767

[B10] Chen B. Cai J. Wei Y. Jiang Z. Desjardins H. E. Adams A. E. (2019). Exosomes are comparable to source adipose stem cells in fat graft retention with up-regulating early inflammation and angiogenesis. *Plast. Reconstr. Surg.* 144 816e–827e. 10.1097/PRS.0000000000006175 31385891

[B11] Chen S. Tang Y. Liu Y. Zhang P. Lv L. Zhang X. (2019). Exosomes derived from miR-375-overexpressing human adipose mesenchymal stem cells promote bone regeneration. *Cell Prolif.* 52:e12669. 10.1111/cpr.12669 31380594 PMC6797519

[B12] Cho B. S. Kim J. O. Ha D. H. Yi Y. W. (2018). Exosomes derived from human adipose tissue-derived mesenchymal stem cells alleviate atopic dermatitis. *Stem Cell Res. Ther.* 9:187. 10.1186/s13287-018-0939-5 29996938 PMC6042362

[B13] Cho J. A. Park H. Lim E. H. Lee K. W. (2012). Exosomes from breast cancer cells can convert adipose tissue-derived mesenchymal stem cells into myofibroblast-like cells. *Int. J. Oncol.* 40 130–138. 10.3892/ijo.2011.1193 21904773

[B14] Choi J. Cha Y. J. Koo J. S. (2018). Adipocyte biology in breast cancer: from silent bystander to active facilitator. *Prog. Lipid Res.* 69 11–20. 10.1016/j.plipres.2017.11.002 29175445

[B15] De Lope L. R. Alcíbar O. L. López A. A. Hergueta-Redondo M. Peinado H. (2018). Tumour–adipose tissue crosstalk: fuelling tumour metastasis by extracellular vesicles. *Philos. Trans. R. Soc. B Biol. Sci.* 373:20160485. 10.1098/rstb.2016.0485 29158314 PMC5717439

[B16] Ecker B. L. Lee J. Y. Sterner C. J. Solomon A. C. Pant D. K. Shen F. (2019). Impact of obesity on breast cancer recurrence and minimal residual disease. *Breast Cancer Res.* 21:41. 10.1186/s13058-018-1087-7 30867005 PMC6416940

[B17] Eming S. A. Martin P. Tomic-Canic M. (2014). Wound repair and regeneration: mechanisms, signaling, and translation. *Sci. Transl. Med.* 6:265sr6. 10.1126/scitranslmed.3009337 25473038 PMC4973620

[B18] Figueroa F. E. Cuenca Moreno J. La Cava A. (2014). Novel approaches to lupus drug discovery using stem cell therapy. Role of mesenchymal-stem-cell-secreted factors. *Expert Opin. Drug Discov.* 9 555–566. 10.1517/17460441.2014.897692 24655067

[B19] Fujiwara O. Prasai A. Perez-Bello D. El Ayadi A. Petrov I. Y. Esenaliev R. O. (2020). Adipose-derived stem cells improve grafted burn wound healing by promoting wound bed blood flow. *Burn. Trauma* 8 635–642. 10.1093/burnst/tkaa009 32346539 PMC7175768

[B20] Gentile P. Casella D. Palma E. Calabrese C. (2019a). Engineered fat graft enhanced with adipose-derived stromal vascular fraction cells for regenerative medicine: clinical, histological and instrumental evaluation in breast reconstruction. *J. Clin. Med.* 8:504. 10.3390/jcm8040504 31013744 PMC6518258

[B21] Gentile P. Piccinno M. Calabrese C. (2019b). Characteristics and potentiality of human adipose-derived stem cells (hASCs) obtained from enzymatic digestion of fat graft. *Cells* 8:282. 10.3390/cells8030282 30934588 PMC6469026

[B22] Goto H. Shimono Y. Funakoshi Y. Imamura Y. Toyoda M. Kiyota N. (2019). Adipose-derived stem cells enhance human breast cancer growth and cancer stem cell-like properties through adipsin. *Oncogene* 38 767–779. 10.1038/s41388-018-0477-8 30177835

[B23] Guo S. Wang T. Zhang S. Chen P. Cao Z. Lian W. (2020). Adipose-derived stem cell-conditioned medium protects fibroblasts at different senescent degrees from UVB irradiation damages. *Mol. Cell. Biochem.* 463 67–78. 10.1007/s11010-019-03630-8 31602539

[B24] Ha D. H. Kim H.-K. Lee J. Kwon H. H. Park G.-H. Yang S. H. (2020). Mesenchymal stem/stromal cell-derived exosomes for immunomodulatory therapeutics and skin regeneration. *Cells* 9:1157. 10.3390/cells9051157 32392899 PMC7290908

[B25] Han Y. Ren J. Bai Y. Pei X. Han Y. (2019). International journal of biochemistry and cell biology exosomes from hypoxia-treated human adipose-derived mesenchymal stem cells enhance angiogenesis through VEGF / VEGF-R. *Int. J. Biochem. Cell Biol.* 109 59–68. 10.1016/j.biocel.2019.01.017 30710751

[B26] Han Y. D. Bai Y. Yan X. L. Ren J. Zeng Q. Li X. D. (2018). Co-transplantation of exosomes derived from hypoxia-preconditioned adipose mesenchymal stem cells promotes neovascularization and graft survival in fat grafting. *Biochem. Biophys. Res. Commun.* 497 305–312. 10.1016/j.bbrc.2018.02.076 29428734

[B27] He L. Zhu C. Jia J. Hao X. Y. Yu X. Y. Liu X. Y. (2020). ADSC-Exos containing MALAT1 promotes wound healing by targeting miR-124 through activating Wnt/β-catenin pathway. *Biosci. Rep.* 40:BSR20192549. 10.1042/BSR20192549 32342982 PMC7214401

[B28] He N. Kong Y. Lei X. Liu Y. Wang J. Xu C. (2018). MSCs inhibit tumor progression and enhance radiosensitivity of breast cancer cells by down-regulating Stat3 signaling pathway. *Cell Death Dis.* 9:1026. 10.1038/s41419-018-0949-3 30297887 PMC6175943

[B29] Henrotin Y. Pesesse L. Sanchez C. (2012). Subchondral bone and osteoarthritis: biological and cellular aspects. *Osteoporos. Int.* 23 847–851. 10.1007/s00198-012-2162-z 23179567

[B30] Hong P. Yang H. Wu Y. Li K. Tang Z. (2019). The functions and clinical application potential of exosomes derived from adipose mesenchymal stem cells: a comprehensive review. *Stem Cell Res. Ther.* 10:242. 10.1186/s13287-019-1358-y 31391108 PMC6686455

[B31] Hong Z. Chen J. Zhang S. Zhao C. Bi M. Chen X. (2019). Intra-articular injection of autologous adipose-derived stromal vascular fractions for knee osteoarthritis: a double-blind randomized self-controlled trial. *Int. Orthop.* 43 1123–1134. 10.1007/s00264-018-4099-0 30109404

[B32] Hu L. Wang J. Zhou X. Xiong Z. Zhao J. Yu R. (2016). Exosomes derived from human adipose mensenchymal stem cells accelerates cutaneous wound healing via optimizing the characteristics of fibroblasts. *Sci. Rep.* 6:32993. 10.1038/srep32993 27615560 PMC5018733

[B33] Hu S. Li Z. Cores J. Huang K. Su T. Dinh P.-U. (2019). Needle-free injection of exosomes derived from human dermal fibroblast spheroids ameliorates skin photoaging. *ACS Nano* 13 11273–11282. 10.1021/acsnano.9b04384 31449388 PMC7032013

[B34] Huang J.-Y. Wang Y.-Y. Lo S. Tseng L.-M. Chen D.-R. Wu Y.-C. (2019). Visfatin mediates malignant behaviors through adipose-derived stem cells intermediary in breast cancer. *Cancers* 12:29. 10.3390/cancers12010029 31861872 PMC7016886

[B35] Kalluri R. LeBleu V. S. (2020). The biology, function, and biomedical applications of exosomes. *Science* 367:eaau6977. 10.1126/science.aau6977 32029601 PMC7717626

[B36] Katsuda T. Tsuchiya R. Kosaka N. Yoshioka Y. Takagaki K. Oki K. (2013). Human adipose tissue-derived mesenchymal stem cells secrete functional neprilysin-bound exosomes. *Sci. Rep.* 3:1197. 10.1038/srep01197 23378928 PMC3561625

[B37] Kim H. Lee B. K. (2020). Anti-inflammatory effect of adipose-derived stromal vascular fraction on osteoarthritic temporomandibular joint synoviocytes. *Tissue Eng. Regen. Med.* 17 351–362. 10.1007/s13770-020-00268-2 32367459 PMC7260348

[B38] Kim K. Fan Y. Lin G. Park Y. K. Pak C. S. Jeong J. H. (2019). synergistic effect of adipose-derived stem cells and fat graft on wrinkles in aged mice. *Plast. Reconstr. Surg.* 143 1637–1646. 10.1097/PRS.0000000000005625 30907792

[B39] Kim W.-S. Park B.-S. Kim H.-K. Park J.-S. Kim K.-J. Choi J.-S. (2008). Evidence supporting antioxidant action of adipose-derived stem cells: protection of human dermal fibroblasts from oxidative stress. *J. Dermatol. Sci.* 49 133–142. 10.1016/j.jdermsci.2007.08.004 17870415

[B40] Kølle S. T. Duscher D. Taudorf M. Fischer-Nielsen A. Svalgaard J. D. Munthe-Fog L. (2020). Ex vivo-expanded autologous adipose tissue-derived stromal cells ensure enhanced fat graft retention in breast augmentation: a randomized controlled clinical trial. *Stem Cells Transl. Med.* [Epub ahead of print] 10.1002/sctm.20-0081 32639099 PMC7581442

[B41] Krastev T. Van Turnhout A. Vriens E. Smits L. Van Der Hulst R. (2019). Long-term follow-up of autologous fat transfer vs conventional breast reconstruction and association with cancer relapse in patients with breast cancer. *Jama Surg.* 154 56–63. 10.1001/jamasurg.2018.3744 30304330 PMC6439860

[B42] Lee H. R. Kim T. H. Oh S. H. Lee J. H. (2018). Prednisolone-loaded coatable polyvinyl alcohol/alginate hydrogel for the treatment of atopic dermatitis. *J. Biomater. Sci. Polym. Ed.* 29 1612–1624. 10.1080/09205063.2018.1477317 29764304

[B43] Lee K. S. Choi J. S. Cho Y. W. (2019). Reprogramming of cancer stem cells into non-tumorigenic cells using stem cell exosomes for cancer therapy. *Biochem. Biophys. Res. Commun.* 512 511–516. 10.1016/j.bbrc.2019.03.072 30905410

[B44] Li L. Ngo H. T. T. Hwang E. Wei X. Liu Y. Liu J. (2019). Conditioned medium from human adipose-derived mesenchymal stem cell culture prevents UVB-induced skin aging in human keratinocytes and dermal fibroblasts. *Int. J. Mol. Sci.* 21:49. 10.3390/ijms21010049 31861704 PMC6981944

[B45] Li W. Liu Y. Zhang P. Tang Y. Zhou M. Jiang W. (2018). Tissue-engineered bone immobilized with human adipose stem cells-derived exosomes promotes bone regeneration. *ACS Appl. Mater. Interfaces* 10 5240–5254. 10.1021/acsami.7b17620 29359912

[B46] Li X. Xie X. Lian W. Shi R. Han S. Zhang H. (2018). Exosomes from adipose-derived stem cells overexpressing Nrf2 accelerate cutaneous wound healing by promoting vascularization in a diabetic foot ulcer rat model. *Exp. Mol. Med.* 50:29. 10.1038/s12276-018-0058-5 29651102 PMC5938041

[B47] Lin R. Wang S. Zhao R. C. (2013). Exosomes from human adipose-derived mesenchymal stem cells promote migration through Wnt signaling pathway in a breast cancer cell model. *Mol. Cell. Biochem.* 383 13–20. 10.1007/s11010-013-1746-z 23812844

[B48] Liu K. Chen C. Zhang H. Chen Y. Zhou S. (2019). Adipose stem cell-derived exosomes in combination with hyaluronic acid accelerate wound healing through enhancing re-epithelialization and vascularization. *Br. J. Dermatol.* 181 854–856. 10.1111/bjd.17984 30953591

[B49] Liu S. Jiang L. Li H. Shi H. Luo H. Zhang Y. (2014). Mesenchymal stem cells prevent hypertrophic scar formation via inflammatory regulation when undergoing apoptosis. *J. Invest. Dermatol.* 134 2648–2657. 10.1038/jid.2014.169 24714203

[B50] Liu X. Yang Y. Li Y. Niu X. Zhao B. Wang Y. (2017). Integration of stem cell-derived exosomes with in situ hydrogel glue as a promising tissue patch for articular cartilage regeneration. *Nanoscale* 9 4430–4438. 10.1039/C7NR00352H 28300264

[B51] Lou G. Chen Z. Zheng M. Liu Y. (2017). Mesenchymal stem cell-derived exosomes as a new therapeutic strategy for liver diseases. *Exp. Mol. Med.* 49:e346. 10.1038/emm.2017.63 28620221 PMC5519012

[B52] Lu Z. Chen Y. Dunstan C. Roohani-Esfahani S. Zreiqat H. (2017). Priming adipose stem cells with tumor necrosis factor-alpha preconditioning potentiates their exosome efficacy for bone regeneration. *Tissue Eng. Part A* 23 1212–1220. 10.1089/ten.tea.2016.0548 28346798

[B53] Ma T. Fu B. Yang X. Xiao Y. Pan M. (2019). Adipose mesenchymal stem cell-derived exosomes promote cell proliferation, migration, and inhibit cell apoptosis via Wnt/β-catenin signaling in cutaneous wound healing. *J. Cell. Biochem.* 120 10847–10854. 10.1002/jcb.28376 30681184

[B54] Maguire G. (2019). The safe and efficacious use of secretome from fibroblasts and adiposederived (but not Bone Marrowderived) mesenchymal stem cells for skin therapeutics. *J. Clin. Aesthet. Dermatol.* 12 E57–E69. PMC6715117 31531174

[B55] Mautner K. Bowers R. Easley K. Fausel Z. Robinson R. (2019). Functional outcomes following microfragmented adipose tissue versus bone marrow aspirate concentrate injections for symptomatic knee osteoarthritis. *Stem Cells Transl. Med.* 8 1149–1156. 10.1002/sctm.18-0285 31328447 PMC6811695

[B56] Mazini L. Rochette L. Amine M. Malka G. (2019). Regenerative capacity of adipose derived stem cells (ADSCs), comparison with mesenchymal stem cells (MSCs). *Int. J. Mol. Sci.* 20:2523. 10.3390/ijms20102523 31121953 PMC6566837

[B57] Mehryab F. Rabbani S. Shahhosseini S. Shekari F. Fatahi Y. Baharvand H. (2020). Exosomes as a next-generation drug delivery system: an update on drug loading approaches, characterization, and clinical application challenges. *Acta Biomater.* 113 42–62. 10.1016/j.actbio.2020.06.036 32622055

[B58] Naderi N. Combellack E. J. Griffin M. Sedaghati T. Javed M. Findlay M. W. (2017). The regenerative role of adipose-derived stem cells (ADSC) in plastic and reconstructive surgery. *Int. Wound J.* 14 112–124. 10.1111/iwj.12569 26833722 PMC7949873

[B59] O’Halloran N. Courtney D. Kerin M. J. Lowery A. J. (2017). Adipose-derived stem cells in novel approaches to breast reconstruction: their suitability for tissue engineering and oncological safety. *Breast Cancer Basic Clin. Res.* 11:117822341772677. 10.1177/1178223417726777 29104428 PMC5562338

[B60] O’Halloran N. Potter S. Kerin M. Lowery A. (2018). Recent advances and future directions in postmastectomy breast reconstruction. *Clin. Breast Cancer* 18 e571–e585. 10.1016/j.clbc.2018.02.004 29572079

[B61] Paduano F. Marrelli M. Amantea M. Rengo C. Rengo S. Goldberg M. (2017). Adipose tissue as a strategic source of mesenchymal stem cells in bone regeneration: a topical review on the most promising craniomaxillofacial applications. *Int. J. Mol. Sci.* 18:2140. 10.3390/ijms18102140 29027958 PMC5666822

[B62] Paolella F. Manferdini C. Gabusi E. Gambari L. Filardo G. Kon E. (2019). Effect of microfragmented adipose tissue on osteoarthritic synovial macrophage factors. *J. Cell. Physiol.* 234 5044–5055. 10.1002/jcp.27307 30187478

[B63] Park A. Park H. Yoon J. Kang D. Kang M.-H. Park Y.-Y. (2019). Priming with Toll-like receptor 3 agonist or interferon-gamma enhances the therapeutic effects of human mesenchymal stem cells in a murine model of atopic dermatitis. *Stem Cell Res. Ther.* 10:66. 10.1186/s13287-019-1164-6 30795812 PMC6387524

[B64] Perez-Hernandez J. Redon J. Cortes R. (2017). Extracellular vesicles as therapeutic agents in systemic lupus erythematosus. *Int. J. Mol. Sci.* 18:717. 10.3390/ijms18040717 28350323 PMC5412303

[B65] Pers Y. M. Quentin J. Feirreira R. Espinoza F. Abdellaoui N. Erkilic N. (2018). Injection of adipose-derived stromal cells in the knee of patients with severe osteoarthritis has a systemic effect and promotes an anti-inflammatory phenotype of circulating immune cells. *Theranostics* 8 5519–5528. 10.7150/thno.27674 30555561 PMC6276295

[B66] Pers Y. M. Ruiz M. Noël D. Jorgensen C. (2015). Mesenchymal stem cells for the management of inflammation in osteoarthritis: state of the art and perspectives. *Osteoarthr. Cartil.* 23 2027–2035. 10.1016/j.joca.2015.07.004 26521749

[B67] Pu C. Liu C. Liang C. Yen Y. Chen S.-H. Jiang-Shieh Y.-F. (2017). Adipose-derived stem cells protect skin flaps against ischemia/reperfusion injury via IL-6 expression. *J. Invest. Dermatol.* 137 1353–1362. 10.1016/j.jid.2016.12.030 28163069

[B68] Qiu H. Liu S. Wu K. Zhao R. Cao L. Wang H. (2020). Prospective application of exosomes derived from adipose-derived stem cells in skin wound healing: a review. *J. Cosmet. Dermatol.* 19 574–581. 10.1111/jocd.13215 31755172

[B69] Ragni E. Perucca Orfei C. De Luca P. Lugano G. Viganò M. Colombini A. (2019). Interaction with hyaluronan matrix and miRNA cargo as contributors for in vitro potential of mesenchymal stem cell-derived extracellular vesicles in a model of human osteoarthritic synoviocytes. *Stem Cell Res. Ther.* 10 1–17. 10.1186/s13287-019-1215-z 30922413 PMC6440078

[B70] Ren L. Song Z. Cai Q. Chen R. Zou Y. Fu Q. (2019). Adipose mesenchymal stem cell-derived exosomes ameliorate hypoxia/serum deprivation-induced osteocyte apoptosis and osteocyte-mediated osteoclastogenesis in vitro. *Biochem. Biophys. Res. Commun.* 508 138–144. 10.1016/j.bbrc.2018.11.109 30473217

[B71] Sah S. K. Agrahari G. Nguyen C. T. Kim Y.-S. Kang K.-S. Kim T.-Y. (2018). Enhanced therapeutic effects of human mesenchymal stem cells transduced with superoxide dismutase 3 in a murine atopic dermatitis-like skin inflammation model. *Allergy* 73 2364–2376. 10.1111/all.13594 30144097

[B72] Sauter M. A. Brett E. Müller C. M. Machens H. G. Duscher D. (2019). Novel assay analyzing tropism between adipose-derived stem cells and breast cancer cells reveals a low oncogenic response. *Breast Care* 14 278–287. 10.1159/000503411 31798387 PMC6883470

[B73] Shi R. Jin Y. Hu W. Lian W. Cao C. Han S. (2020). Exosomes derived from mmu_circ_0000250-modified adipose-derived mesenchymal stem cells promote wound healing in diabetic mice by inducing miR-128-3p/SIRT1-mediated autophagy. *Am. J. Physiol. Physiol.* 318 C848–C856. 10.1152/ajpcell.00041.2020 32159361

[B74] Shin K.-O. Ha D. H. Kim J. O. Crumrine D. A. Meyer J. M. Wakefield J. S. (2020). Exosomes from human adipose tissue-derived mesenchymal stem cells promote epidermal barrier repair by inducing de novo synthesis of ceramides in atopic dermatitis. *Cells* 9:680. 10.3390/cells9030680 32164386 PMC7140723

[B75] Shin T.-H. Kim H.-S. Choi S. Kang K.-S. (2017). Mesenchymal stem cell therapy for inflammatory skin diseases: clinical potential and mode of action. *Int. J. Mol. Sci.* 18:244. 10.3390/ijms18020244 28125063 PMC5343781

[B76] Shukla L. Yuan Y. Shayan R. Greening D. W. Karnezis T. (2020). Fat therapeutics: the clinical capacity of adipose-derived stem cells and exosomes for human disease and tissue regeneration. *Front. Pharmacol.* 11:158. 10.3389/fphar.2020.00158 32194404 PMC7062679

[B77] Silva M. M. A. Kokai L. E. Donnenberg V. S. Fine J. L. Marra K. G. Donnenberg A. D. (2019). Oncologic safety of fat grafting for autologous breast reconstruction in an animal model of residual breast cancer. *Plast. Reconstr. Surg.* 143 103–112. 10.1097/PRS.0000000000005085 30589782 PMC8462977

[B78] Sorkin A. Heller L. Landau G. Sherf M. Hartstein M. E. Hadad E. (2020). Inferiorly based preauricular flap for anterior ear reconstruction. *Ann. Plast. Surg.* 84 394–396. 10.1097/SAP.0000000000002124 31904646

[B79] Spasovski D. Spasovski V. Bašèareviæ Z. Stojiljkoviæ M. Vreæa M. Anðelkoviæ M. (2018). Intra-articular injection of autologous adipose-derived mesenchymal stem cells in the treatment of knee osteoarthritis. *J. Gene Med.* 20:e3002. 10.1002/jgm.3002 29243283

[B80] Strong A. L. Bowles A. C. Wise R. M. Morand J. P. Dutreil M. F. Gimble J. M. (2016). Human adipose stromal/stem cells from obese donors show reduced efficacy in halting disease progression in the experimental autoimmune encephalomyelitis model of multiple sclerosis. *Stem Cells* 34 614–626. 10.1002/stem.2272 26700612 PMC4803617

[B81] Strong A. L. Rubin J. P. Kozlow J. H. Cederna P. S. (2019). Fat grafting for the treatment of scleroderma. *Plast. Reconstr. Surg.* 144 1498–1507. 10.1097/PRS.0000000000006291 31764674

[B82] Tan S. S. Loh W. (2017). The utility of adipose-derived stem cells and stromal vascular fraction for oncologic soft tissue reconstruction: is it safe? A matter for debate. *Surgeon* 15 186–189. 10.1016/j.surge.2016.09.010 27810224

[B83] Teufelsbauer M. Rath B. Moser D. Haslik W. Huk I. Hamilton G. (2019). Interaction of adipose-derived stromal cells with breast cancer cell lines. *Plast. Reconstr. Surg.* 144 207E–217E. 10.1097/PRS.0000000000005839 31348343

[B84] Tofiño-Vian M. Guillén M. I. Pérez Del Caz M. D. Castejón M. A. Alcaraz M. J. (2017). Extracellular vesicles from adipose-derived mesenchymal stem cells downregulate senescence features in osteoarthritic osteoblasts. *Oxid. Med. Cell. Longev.* 2017:7197598. 10.1155/2017/7197598 29230269 PMC5694590

[B85] Tofiño-Vian M. Guillén M. I. Pérez Del Caz M. D. Silvestre A. Alcaraz M. J. (2018). Microvesicles from human adipose tissue-derived mesenchymal stem cells as a new protective strategy in osteoarthritic chondrocytes. *Cell. Physiol. Biochem.* 47 11–25. 10.1159/000489739 29763932

[B86] Togliatto G. Dentelli P. Gili M. Gallo S. Deregibus C. Biglieri E. (2016). Obesity reduces the pro-angiogenic potential of adipose tissue stem cell-derived extracellular vesicles (EVs) by impairing miR-126 content: impact on clinical applications. *Int. J. Obes.* 40 102–111. 10.1038/ijo.2015.123 26122028 PMC4722244

[B87] Toh W. S. Zhang B. I. N. Lai R. C. Lim S. K. (2018). Immune regulatory targets of mesenchymal stromal cell exosomes/small extracellular vesicles in tissue regeneration. *Cytotherapy* 20 1419–1426. 10.1016/j.jcyt.2018.09.008 30352735

[B88] Tran T. D. X. Wu C. Dubey N. K. Deng Y. Su C.-W. Pham T. T. (2019). Time- and Kellgren–Lawrence grade-dependent changes in intra-articularly transplanted stromal vascular fraction in osteoarthritic patients. *Cells* 8:308. 10.3390/cells8040308 30987218 PMC6523621

[B89] Villatoro A. J. Alcoholado C. Martín-Astorga M. C. Fernández V. Cifuentes M. Becerra J. (2019). Comparative analysis and characterization of soluble factors and exosomes from cultured adipose tissue and bone marrow mesenchymal stem cells in canine species. *Vet. Immunol. Immunopathol.* 208 6–15. 10.1016/j.vetimm.2018.12.003 30712794

[B90] Vizoso F. J. Eiro N. Cid S. Schneider J. Perez-Fernandez R. (2017). Mesenchymal stem cell secretome: toward cell-free therapeutic strategies in regenerative medicine. *Int. J. Mol. Sci.* 18:1852. 10.3390/ijms18091852 28841158 PMC5618501

[B91] Wang C. Wang M. Xu T. Zhang X. Lin C. Gao W. (2019). Engineering bioactive self-healing antibacterial exosomes hydrogel for promoting chronic diabetic wound healing and complete skin regeneration. *Theranostics* 9 65–76. 10.7150/thno.29766 30662554 PMC6332800

[B92] Wang M. Wang C. Chen M. Xi Y. Cheng W. Mao C. (2019). Efficient angiogenesis-based diabetic wound healing/skin reconstruction through bioactive antibacterial adhesive ultraviolet shielding nanodressing with exosome release. *ACS Nano* 13 10279–10293. 10.1021/acsnano.9b03656 31483606

[B93] Wang J. Guo X. Kang Z. Qi L. Yang Y. Wang J. (2020). Roles of exosomes from mesenchymal stem cells in treating osteoarthritis. *Cell. Reprogram.* 22 107–117. 10.1089/cell.2019.0098 32364765

[B94] Wang Q. Zhang N. Hu L. Xi Y. Mi W. Ma Y. (2020). Integrin β1 in adipose-derived stem cells accelerates wound healing via activating PI3K/AKT pathway. *Tissue Eng. Regen. Med.* 17 183–192. 10.1007/s13770-019-00229-4 32200515 PMC7105588

[B95] Wang L. Hu L. Zhou X. Xiong Z. Zhang C. Shehada H. M. A. (2017). Exosomes secreted by human adipose mesenchymal stem cells promote scarless cutaneous repair by regulating extracellular matrix remodelling. *Sci. Rep.* 7:13321. 10.1038/s41598-017-12919-x 29042658 PMC5645460

[B96] Wang T. Jiang A. Guo Y. Tan Y. Tang G. Mai M. (2013). Deep sequencing of the transcriptome reveals inflammatory features of porcine visceral adipose tissue. *Int. J. Biol. Sci.* 9 550–556. 10.7150/ijbs.6257 23781149 PMC3683940

[B97] Wang W. Wu C. Jin H. (2018). Exosomes in chronic inflammatory skin diseases and skin tumors. *Exp. Dermatol.* 28 213–218. 10.1111/exd.13857 30537027

[B98] Woo C. H. Kim H. K. Jung G. Y. Jung Y. J. Lee K. S. Yun Y. E. (2020). Small extracellular vesicles from human adipose-derived stem cells attenuate cartilage degeneration. *J. Extracell. Vesicles* 9:1735249. 10.1080/20013078.2020.1735249 32284824 PMC7144299

[B99] Wu Q. Li J. Li Z. Sun S. Zhu S. Wang L. (2019). Exosomes from the tumour-adipocyte interplay stimulate beige/brown differentiation and reprogram metabolism in stromal adipocytes to promote tumour progression. *J. Exp. Clin. Cancer Res.* 38:223. 10.1186/s13046-019-1210-3 31138258 PMC6537177

[B100] Wu Y. Peng Y. Gao D. Feng C. Yuan X. Li H. (2015). Mesenchymal stem cells suppress fibroblast proliferation and reduce skin fibrosis through a TGF-β3-dependent activation. *Int. J. Low. Extrem. Wounds* 14 50–62. 10.1177/1534734614568373 25858630

[B101] Xiao S. Liu Z. Yao Y. Wei Z. R. Wang D. Deng C. (2019). Diabetic human adipose-derived stem cells accelerate pressure ulcer healing by inducing angiogenesis and neurogenesis. *Stem Cells Dev.* 28 319–328. 10.1089/scd.2018.0245 30608025

[B102] Xiong J. Liu Z. Wu M. Sun M. Xia Y. Wang Y. (2020). Comparison of proangiogenic effects of adipose-derived stem cells and foreskin fibroblast exosomes on artificial dermis prefabricated flaps. *Stem Cells Int.* 2020:5293850. 10.1155/2020/5293850 32089706 PMC7013349

[B103] Xu P. Yu Q. Huang H. Zhang W. J. Li W. (2018). nanofat increases dermis thickness and neovascularization in photoaged nude mouse skin. *Aesthetic Plast. Surg.* 42 343–351. 10.1007/s00266-018-1091-4 29380024

[B104] Yang C. Luo L. Bai X. Shen K. Liu K. Wang J. (2020). Highly-expressed micoRNA-21 in adipose derived stem cell exosomes can enhance the migration and proliferation of the HaCaT cells by increasing the MMP-9 expression through the PI3K/AKT pathway. *Arch. Biochem. Biophys.* 681:108259. 10.1016/j.abb.2020.108259 31926164

[B105] Yang Z. Wei Z. Wu X. Yang H. (2018). Screening of exosomal miRNAs derived from subcutaneous and visceral adipose tissues: determination of targets for the treatment of obesity and associated metabolic disorders. *Mol. Med. Rep.* 18 3314–3324. 10.3892/mmr.2018.9312 30066923 PMC6102639

[B106] Yoshimura K. Sato K. Aoi N. Kurita M. Hirohi T. Harii K. (2008). Cell-assisted lipotransfer for cosmetic breast augmentation: supportive use of adipose-derived stem/stromal cells. *Aesthet. Plast. Surg.* 32 48–55. 10.1007/s00266-007-9019-4 17763894 PMC2175019

[B107] Yucel E. Alagoz M. S. Eren G. G. Yasar E. K. Izmirli H. H. Duruksu G. (2016). Use of adipose-derived mesenchymal stem cells to increase viability of composite grafts. *J. Craniofac. Surg.* 27 1354–1360. 10.1097/SCS.0000000000002707 27258717

[B108] Zhang B. Yang Y. Xiang L. Zhao Z. Ye R. (2019). Adipose-derived exosomes: a novel adipokine in obesity-associated diabetes. *J. Cell. Physiol.* 234 16692–16702. 10.1002/jcp.28354 30807657

[B109] Zhang R. Ma J. Han J. Zhang W. Ma J. (2019). Mesenchymal stem cell related therapies for cartilage lesions and osteoarthritis. *Am. J. Transl. Res.* 11 6275–6289. 31737182 PMC6834499

[B110] Zhang W. Bai X. Zhao B. Li Y. Zhang Y. Li Z. (2018). Cell-free therapy based on adipose tissue stem cell-derived exosomes promotes wound healing via the PI3K/Akt signaling pathway. *Exp. Cell Res.* 370 333–342. 10.1016/j.yexcr.2018.06.035 29964051

[B111] Zhao C. Chen J. Y. Peng W. M. Yuan B. Bi Q. Xu Y. J. (2020). Exosomes from adipose-derived stem cells promote chondrogenesis and suppress inflammation by upregulating miR-145 and miR-221. *Mol. Med. Rep.* 21 1881–1889. 10.3892/mmr.2020.10982 32319611 PMC7057766

[B112] Zhao H. Shang Q. Pan Z. Bai Y. Li Z. Zhang H. (2018). Exosomes from adipose-derived stem cells attenuate adipose inflammation and obesity through polarizing M2 macrophages and beiging in white adipose tissue. *Diabetes* 67 235–247. 10.2337/db17-0356 29133512

[B113] Zhu C. T. Li T. Hu Y. H. Zou M. Guo Q. Qu X. W. (2017). Exosomes secreted by mice adipose-derived stem cells after low-level laser irradiation treatment reduce apoptosis of osteocyte induced by hypoxia. *Eur. Rev. Med. Pharmacol. Sci.* 21 5562–5570. 10.26355/eurrev_201712_13993 29271987

[B114] Zhu Y. Zhang J. Hu X. Wang Z. Wu S. Yi Y. (2020). Supplementation with extracellular vesicles derived from adipose-derived stem cells increases fat graft survival and browning in mice: a cell-free approach to construct beige fat from white fat grafting. *Plast. Reconstr. Surg.* 145 1183–1195. 10.1097/PRS.0000000000006740 32332538

[B115] Zuk P. A. Zhu M. Mizuno H. Huang J. Futrell J. W. Katz A. J. (2001). Multilineage cells from human adipose tissue: implications for cell-based therapies. *Tissue Eng.* 7 211–228. 10.1089/107632701300062859 11304456

